# Supplementary material for: Co-Suppression of NbClpC1 and NbClpC2, Encoding Clp Protease Chaperons, Elicits Significant Changes in the Metabolic Profile of Nicotiana benthamiana
Source: Plants (Basel). 2020 Feb 18;9(2):259. doi: 10.3390/plants9020259 (PMC7076384; doi:10.3390/plants9020259)
Supplement: Supplementary file 1 [file plants-09-00259-s001.zip › Supplementary_data_02-10-20.docx]

**Supplementary Materials**


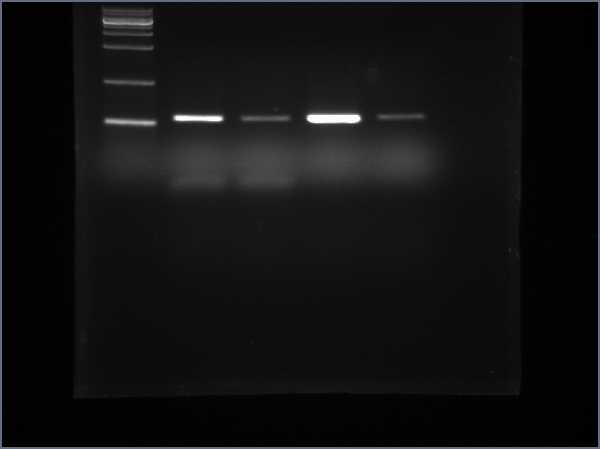

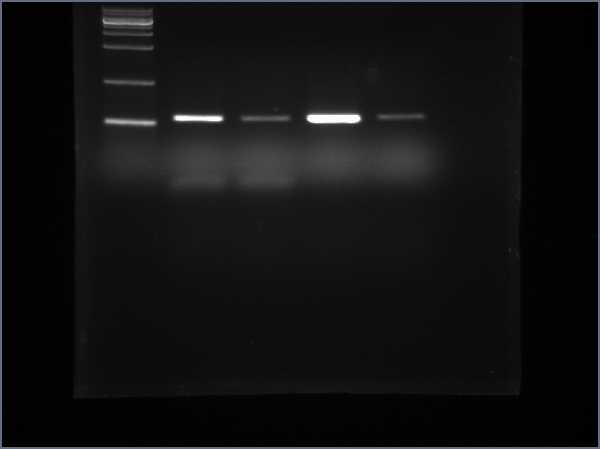

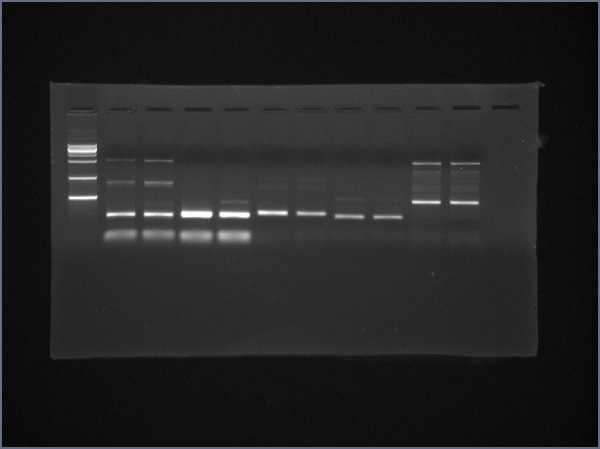


***NbClpC1***

***NbClpC2***

***NbActin***

**Control**

**ClpC**

**Figure S1.** *NbClpC1/C2* co-suppression in *N. benthamiana*. Semi-quantitative RT-PCR analysis for identification of the silencing of *NbClpC1* and *NbClpC2* expression separately. The actin gene was used to identify the equal use of total RNA.

**MATERIALS AND METHODS**

**Materials**

*Nicotiana benthamiana* sample was sent from Yeungnam University to HMT under freezing condition.

Sample information are shown in Table 1.

| Name | Amount (mg) | Group |
| --- | --- | --- |
| NBC-1 | 50 | Control |
| NBC-2 | 50 |  |
| NBC-3 | 50 |  |
| NBT-1 | 50 | Treatment |
| NBT-2 | 50 |  |
| NBT-3 | 50 |  |

**Sample Preparation**

After receipt of samples, 50 μL of Milli-Q water was added to the extract from *Nicotiana benthamiana*. They were subject to CE-TOFMS analysis.

**CE-TOFMS ANALYSIS**

Metabolome analysis (Cation and Anion Mode) was performed in the following conditions. Judging from peak shapes and intensities, they were diluted by 20% for CE-TOFMS analysis in Anion mode.

**Cationic Metabolites (Cation Mode)**

**Device**

Agilent CE-TOFMS system（Agilent Technologies, Inc）Machine No. 3

Capillary: Fused silica capillary i.d. 50 μm × 80 cm

**Analytical Condition**

Run buffer: Cation Buffer Solution (p/n: H3301-1001)

Rinse buffer: Cation Buffer Solution (p/n: H3301-1001)

Sample injection: Pressure injection 50 mbar, 10 sec

CE voltage: Positive, 27 kV

MS ionization: ESI Positive

MS capillary voltage: 4,000 V

MS scan range: *m/z* 50-1,000

Sheath liquid: HMT Sheath Liquid (p/n: H3301-1020)

**Anionic Metabolites (Anion Mode)**

**Device**

Agilent CE-TOFMS system（Agilent Technologies, Inc）Machine No. 5

Capillary: Fused silica capillary i.d. 50 μm × 80 cm

**Analytical Condition**

Run buffer: Anion Buffer Solution (p/n : H3302-1021)

Rinse buffer: Anion Buffer Solution (p/n : H3302-1022)

Sample injection: Pressure injection 50 mbar, 25 sec

CE voltage: Positive, 30 kV

MS ionization: ESI Negative

MS capillary voltage: 3,500 V

MS scan range: *m/z* 50-1,000

Sheath liquid: HMT Sheath Liquid (p/n: H3301-1020)

**DATA PROCESSING AND ANALYSIS**

**Data Processing**

Peaks detected in CE-TOFMS analysis were extracted using the automatic integration software (MasterHands ver. 2.9.0.9, Keio University-developed software). Peak information including *m/z*, migration time (MT) and area was obtained. Peak area was converted into relative peak area according to the following equation. Each peak was aligned according to similar migration time on CE and *m*/*z* value determined by TOFMS.


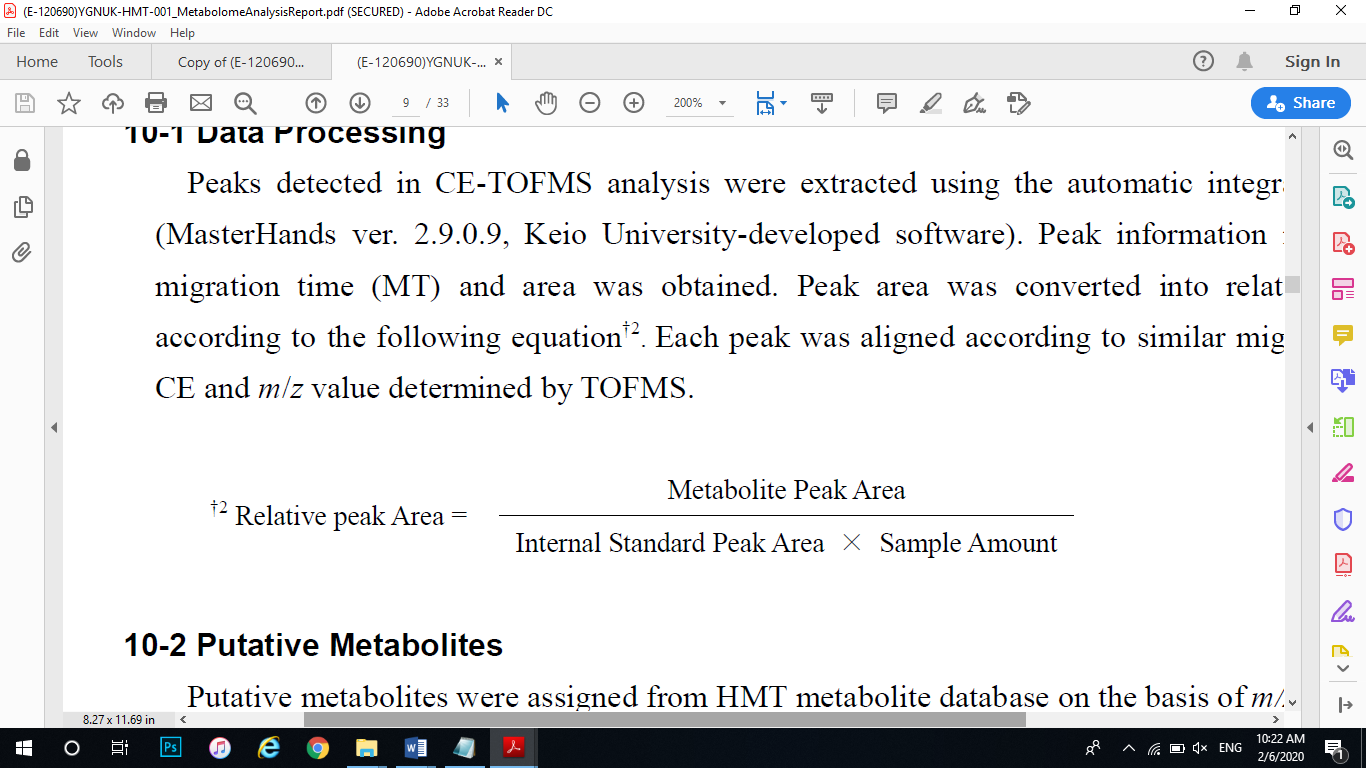


**Putative Metabolites**

Putative metabolites were assigned from HMT metabolite database on the basis of *m/z* and MT. The tolerance was ±0.5 min in MT and ±10 ppm in *m/z*.


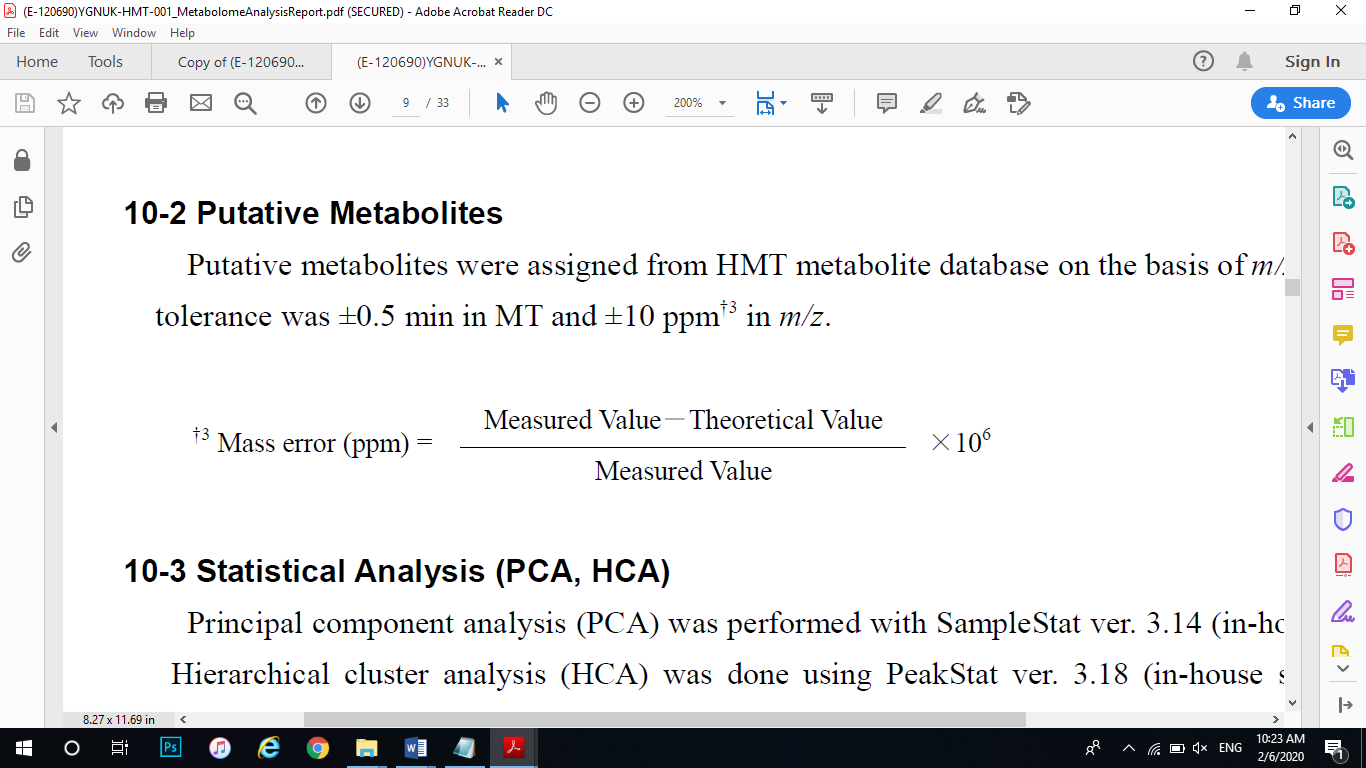


**Statistical Analysis (PCA, HCA)**

Principal component analysis (PCA) was performed with SampleStat ver. 3.14 (in-house software). Hierarchical cluster analysis (HCA) was done using PeakStat ver. 3.18 (in-house software).

**Plotting on Pathway Map**

Detected metabolites were plotted on the map (Figure 1 to 9) using VANTED (Visualization and Analysis of Networks containing Experimental Data). The pathway map in VANTED was made on the basis of metabolic pathway in human.

**Quantitative Estimation of HMT Standard Metabolites**

Quantitative estimation was performed in 108 metabolites including intermediates in glycolysis, TCA cycle, amino acids and nucleic acid. Concentrations of the metabolites were calculated by the normalization with peak area of internal standard. Standard curve for each metabolite was obtained by single-point at 100 μM standard metabolites.


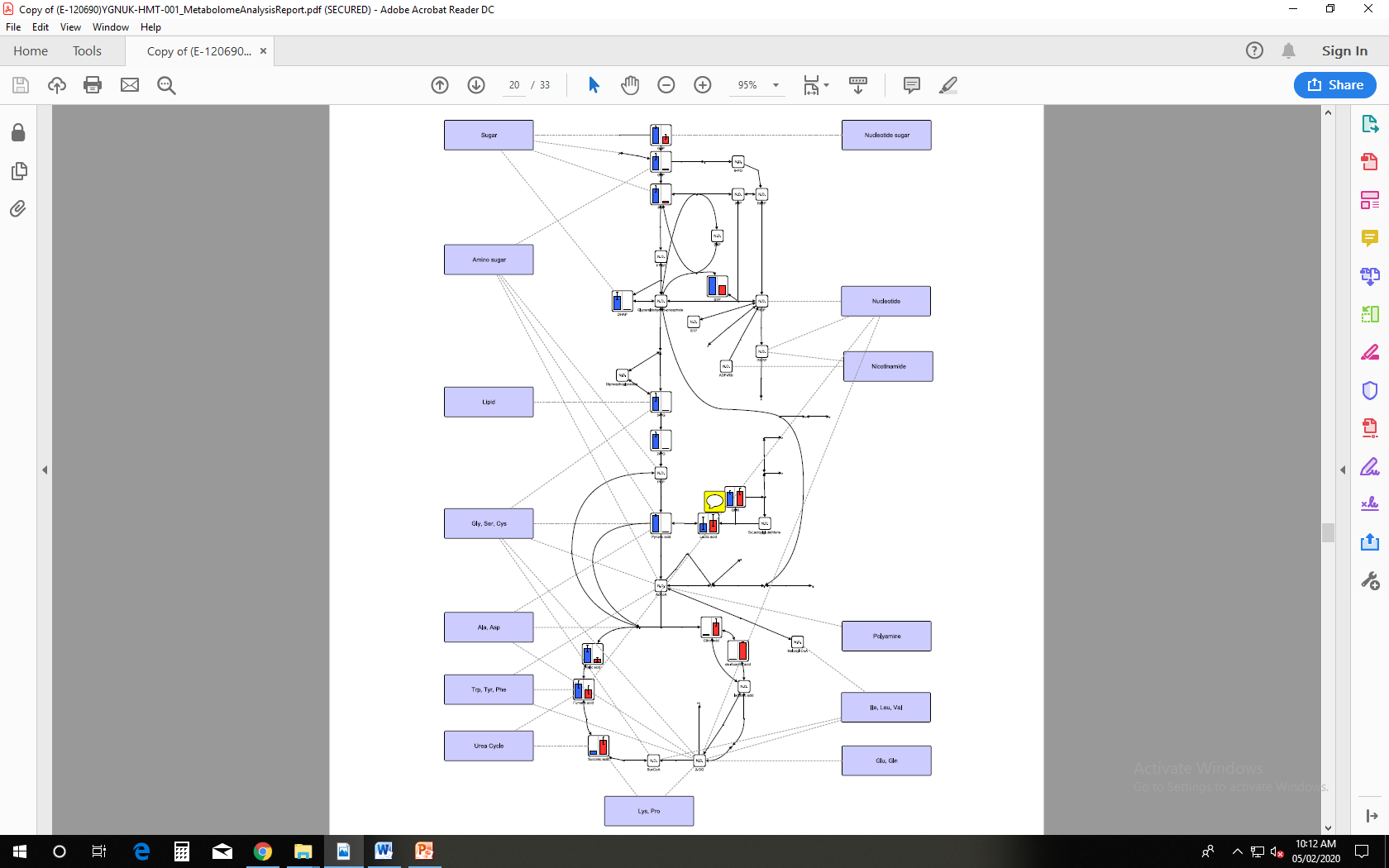


**Figure S2. Pathway Map (Glycolysis, Pentose Phosphate Pathway, TCA Cycle)** In HMT Standard Metabolites, detected metabolites in this study are plotted on the pathway map. Blue and Red vertical bars show Control and Treatment, respectively. N.D.: Not Detected.


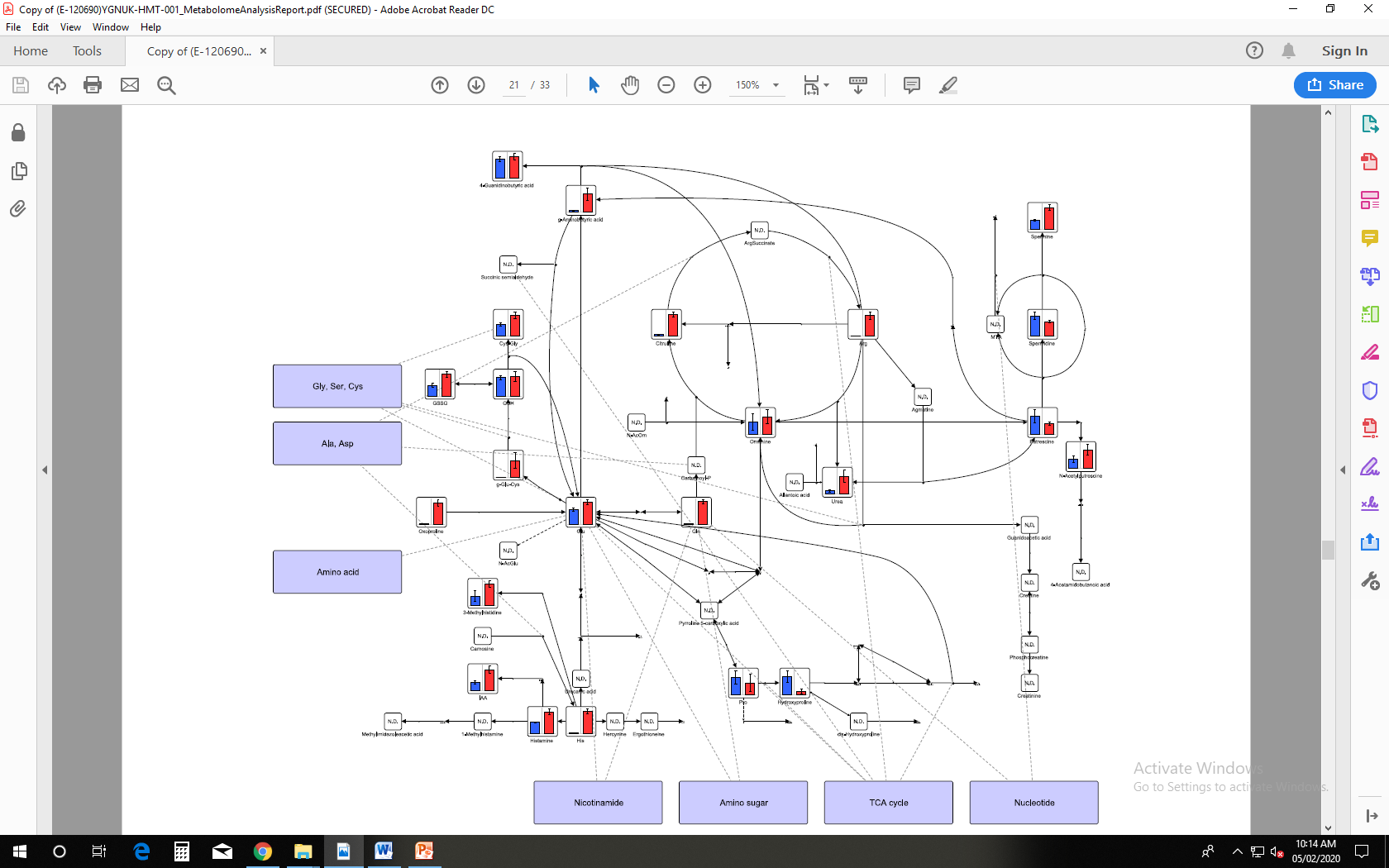


**Figure S3. Pathway Map (Urea Cycle, Glu, Gln, His, Pro)** In HMT Standard Metabolites, detected metabolites in this study are plotted on the pathway map. Blue and Red vertical bars show Control and Treatment, respectively. N.D.: Not Detected.


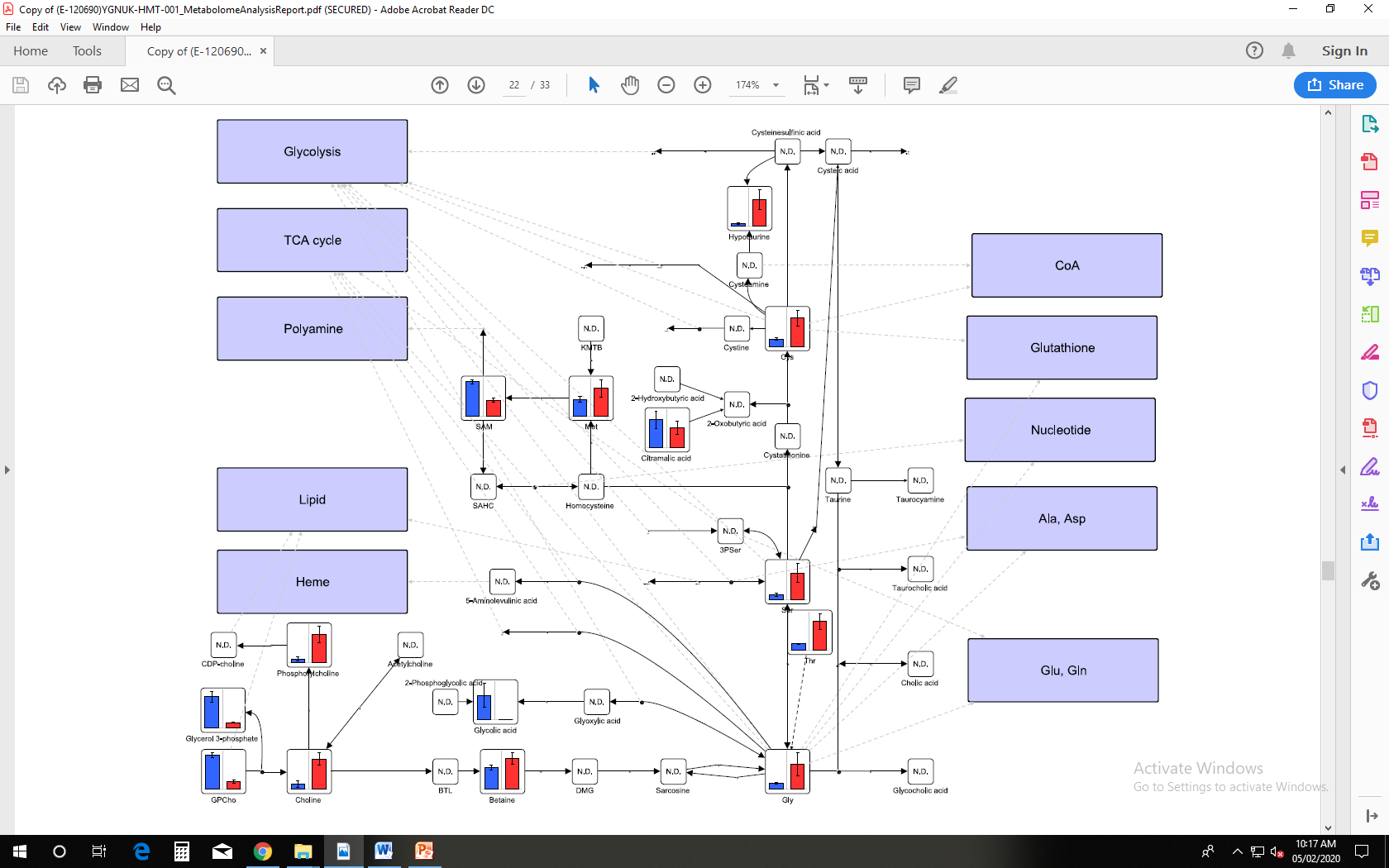


**Figure S4. Pathway Map (Gly, Ser, Cys)** In HMT Standard Metabolites, detected metabolites in this study are plotted on the pathway map. Blue and Red vertical bars show Control and Treatment, respectively. N.D.: Not Detected.


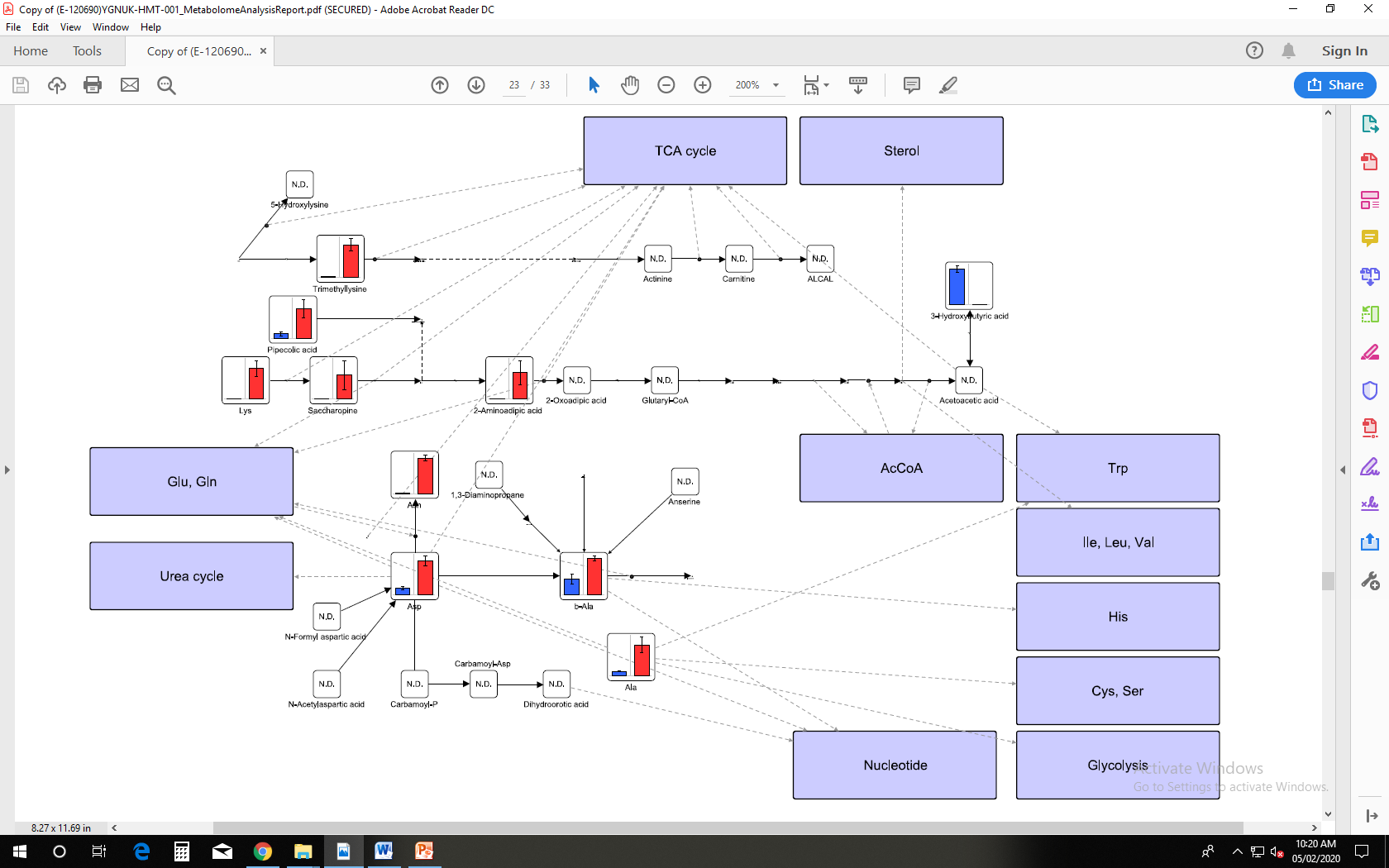


**Figure S5. Pathway Map (Asp, Ala, Lys)** In HMT Standard Metabolites, detected metabolites in this study are plotted on the pathway map. Blue and Red vertical bars show Control and Treatment, respectively. N.D.: Not Detected.


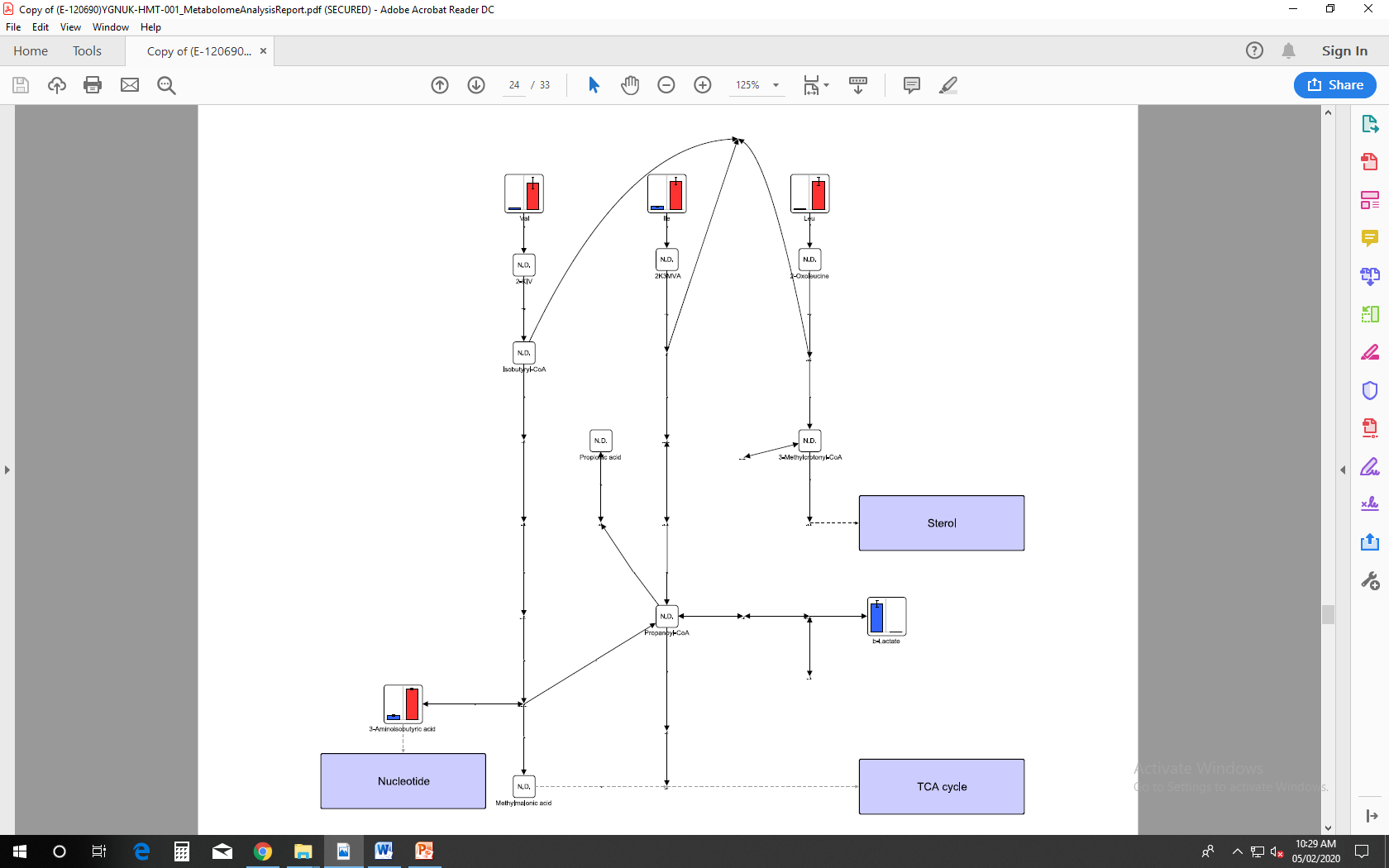


**Figure S6. Pathway Map (Branched-Chain Amino Acids)** In HMT Standard Metabolites, detected metabolites in this study are plotted on the pathway map. Blue and Red vertical bars show Control and Treatment, respectively. N.D.: Not Detected.


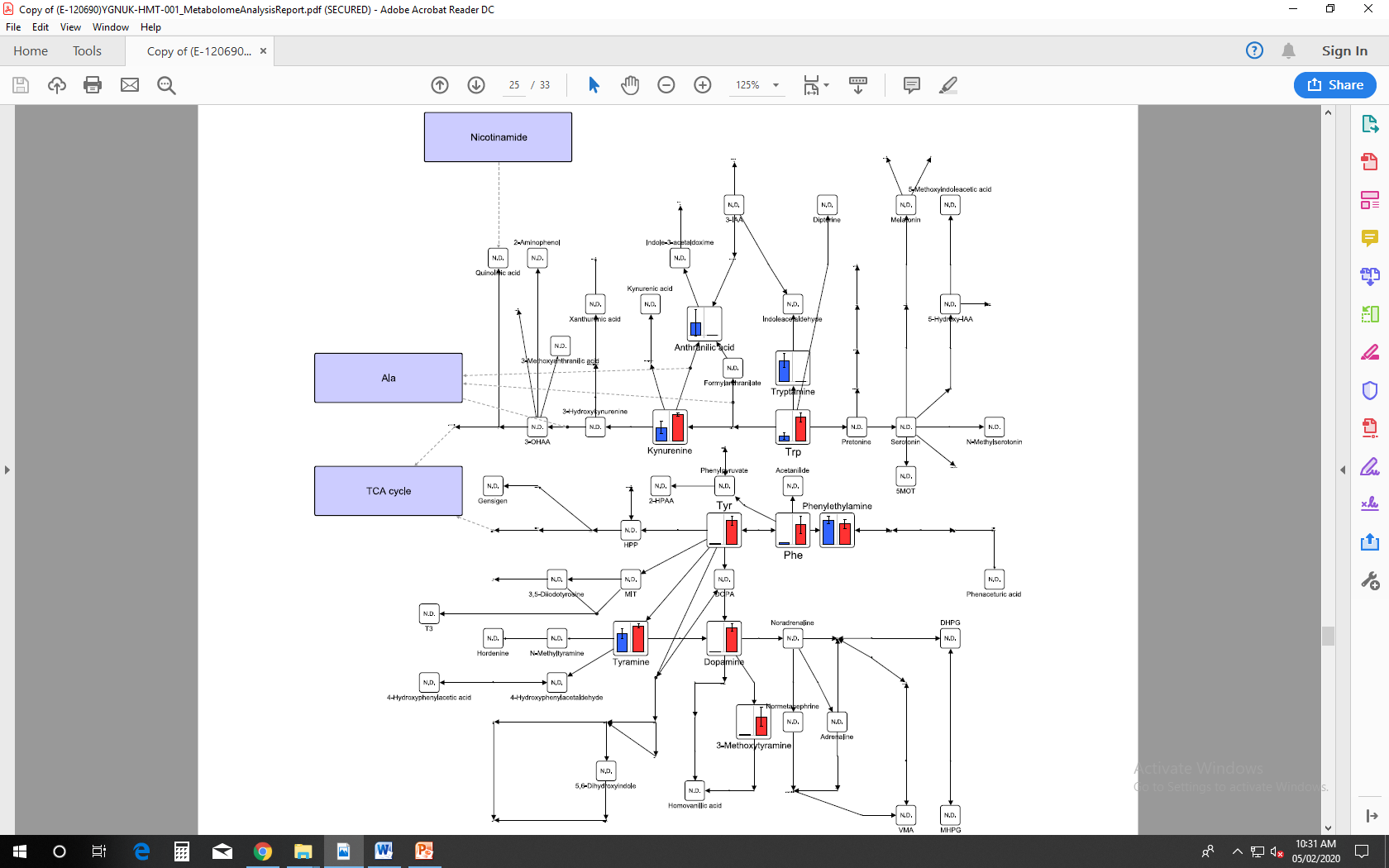


**Figure S7. Pathway Map (Aromatic Amino Acids)** In HMT Standard Metabolites, detected metabolites in this study are plotted on the pathway map. Blue and Red vertical bars show Control and Treatment, respectively. N.D.: Not Detected.


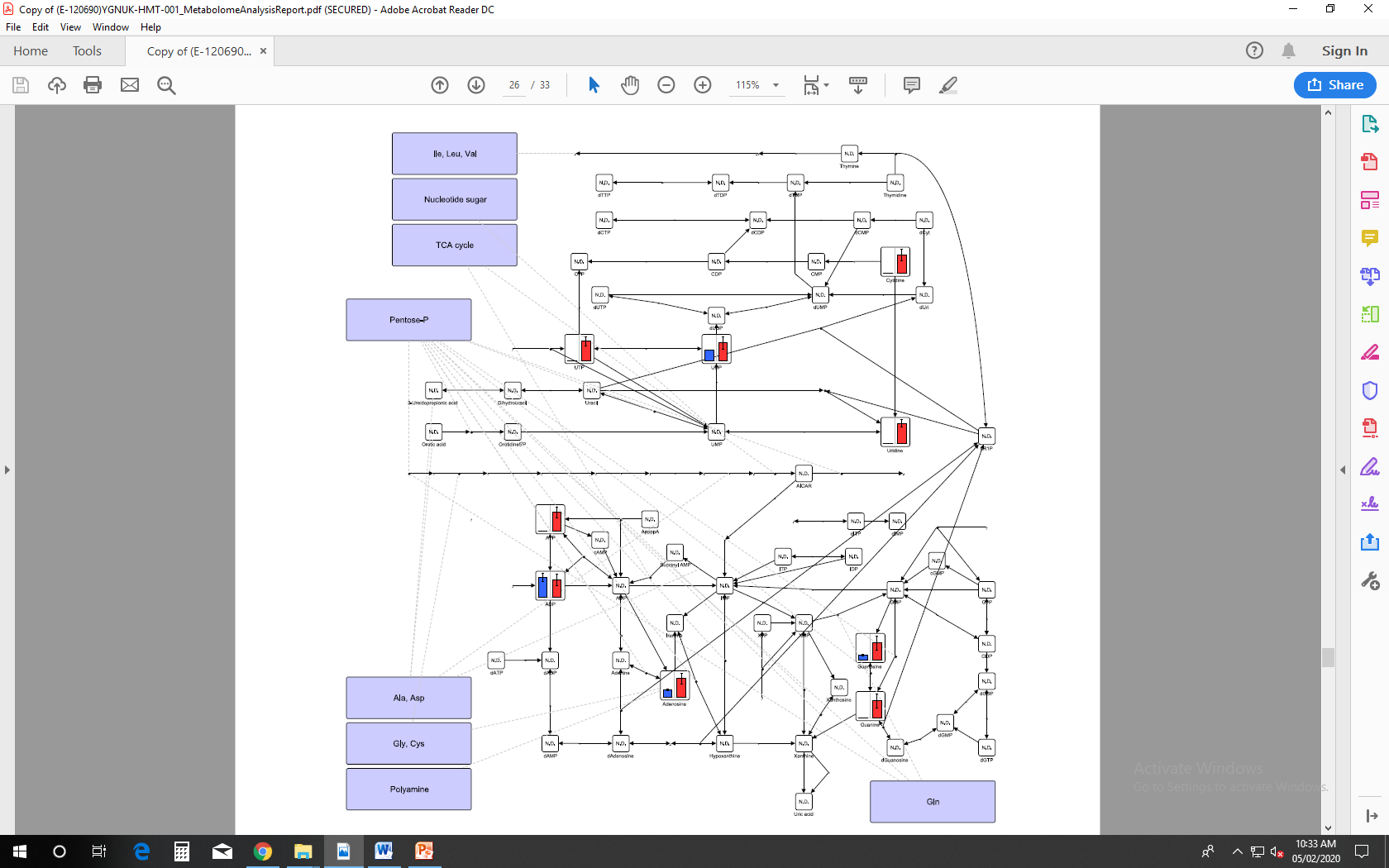


**Figure S8. Pathway Map (Purines, Pyrimidines)** In HMT Standard Metabolites, detected metabolites in this study are plotted on the pathway map. Blue and Red vertical bars show Control and Treatment, respectively. N.D.: Not Detected.


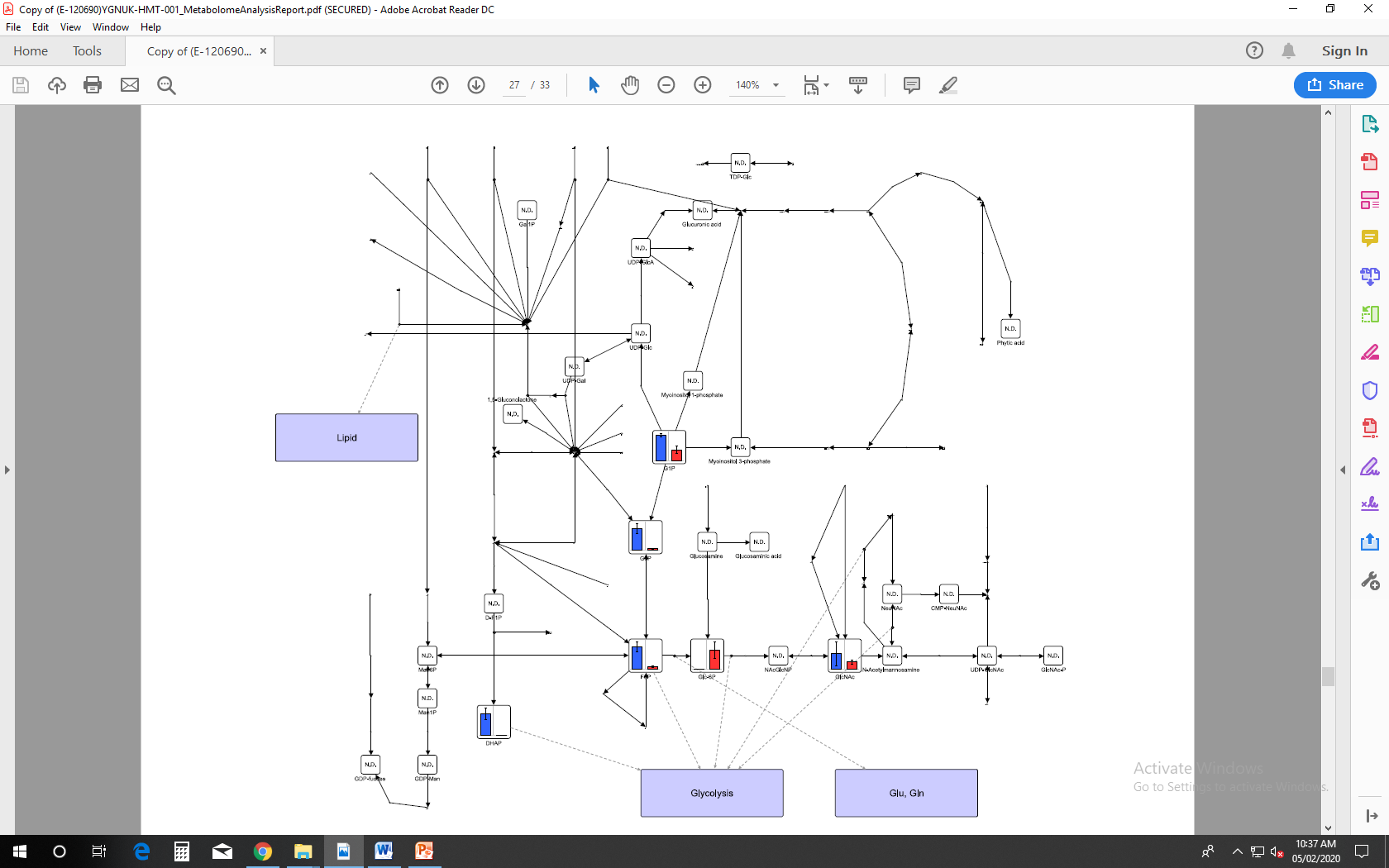


**Figure S9. Pathway Map (Other Sugars)** In HMT Standard Metabolites, detected metabolites in this study are plotted on the pathway map. Blue and Red vertical bars show Control and Treatment, respectively. N.D. : Not Detected.


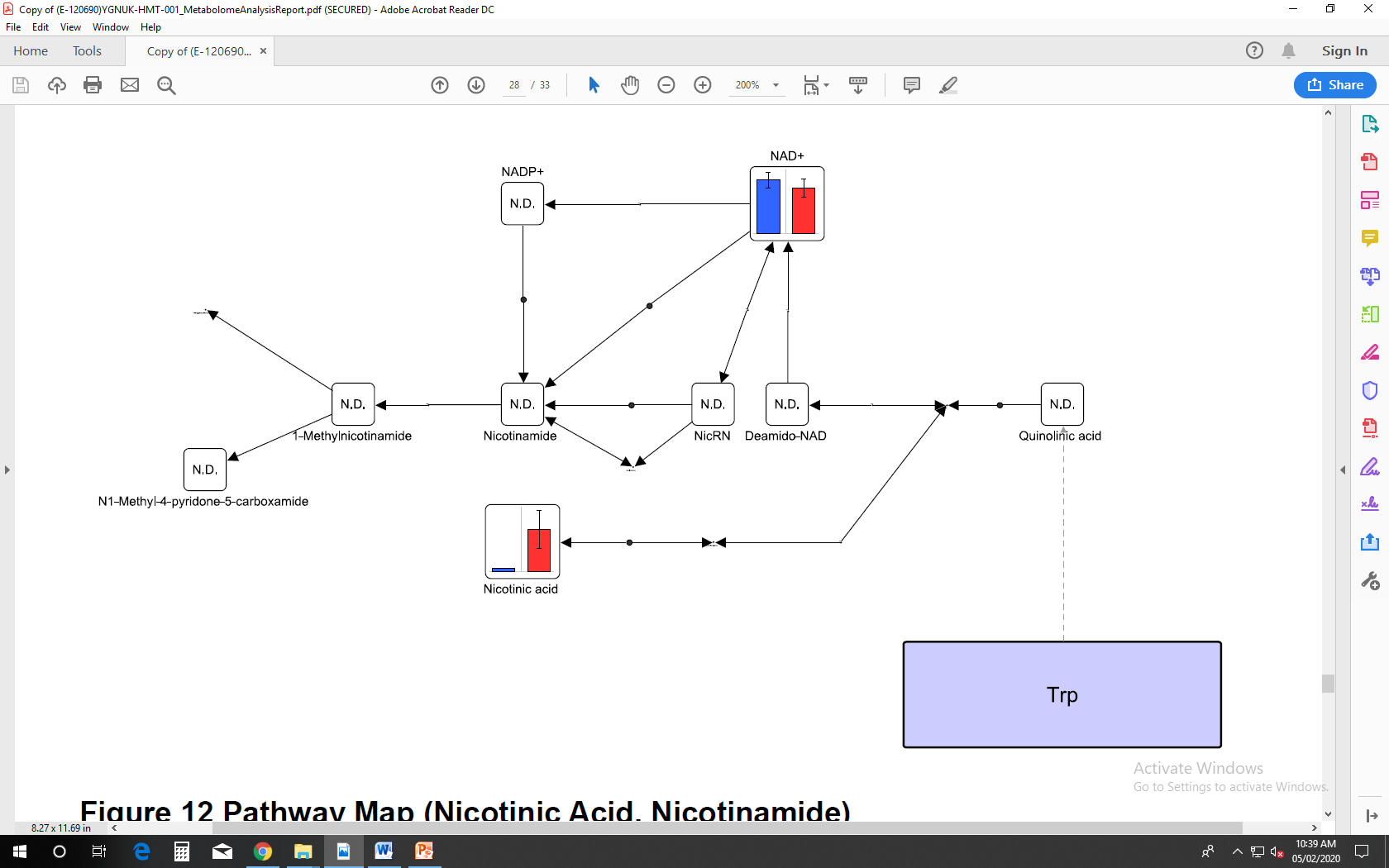


**Figure S10. Pathway Map (Nicotinic Acid, Nicotinamide)** In HMT Standard Metabolites, detected metabolites in this study are plotted on the pathway map. Blue and Red vertical bars show Control and Treatment, respectively. N.D. : Not Detected.

**Table S1. Intracellular metabolites of glycolysis and pentose phosphate pathway detected from Control or *NbClpC1*/*C2* co-suppressed plants (ClpC) using CE-TOF-MS**

| **ID** | **HMT DB^†^ Compound name** | **m/z** | **MT** |  | | | | **Comparative analysis** | |
| --- | --- | --- | --- | --- | --- | --- | --- | --- | --- |
|  |  |  |  | **Control** | | **ClpC** | | **Control vs ClpC** | |
|  |  |  |  | **Mean** | **Standard deviation** | **mean** | **Standard deviation** | **Ratio^¶^** | ***p*-value^\|\|^** |
| A_0005 | Lactic acid | 89.025 | 11.123 | 0.001718 | 0.001240 | 0.002277 | 0.001184 | 1.3 | 0.603 |
| A_0050 | Sedoheptulose 7-phosphate | 289.031 | 9.929 | 0.000059 | N.A. | 0.000031 | N.A. | 0.5 | N.A. |
| A_0047 | Fructose 6-phosphate | 259.021 | 10.252 | 0.000465 | 0.000106 | 0.000047 | 0.000009 | 0.1 | 0.020* |
| A_0046 | Glucose 6-phosphate | 259.021 | 10.155 | 0.002120 | 0.000502 | 0.000171 | 0.000040 | 0.1 | 0.021* |
| A_0003 | Pyruvic acid | 87.008 | 13.427 | 0.000668 | 0.000027 | N.D. | N.A. | <1 | N.A. |
| A_0023 | Dihydroxyacetone phosphate | 168.990 | 13.136 | 0.000215 | 0.000059 | N.D. | N.A. | <1 | N.A. |
| A_0031 | 2-Phosphoglyceric acid | 184.985 | 20.188 | 0.000202 | 0.000033 | N.D. | N.A. | <1 | N.A. |
| A_0032 | 3-Phosphoglyceric acid | 184.985 | 20.627 | 0.001736 | 0.000523 | N.D. | N.A. | <1 | N.A. |

| ID consists of analysis mode and number. 'C' and 'A' showed cation and anion modes, respectively. | | | | |
| --- | --- | --- | --- | --- |
| N.D.: Not Detected. The metabolite which was below detection limits. | |  |  |  |
| N.A.: Not Available. The calculation was not possible. |  |  |  |  |
| ^†^Putative metabolites which were assigned on the basis of m/z and MT | |  |  |  |
| ^¶^In ratio calculation, the latter was denominator. |  |  |  |  |
| ^\|\|^*p*-value in Welch's t-test.*<0.05, **<0.01, ***<0.001 |  |  |  |  |
| They were sorted by the ratio of ClpC to Control in descending order. | | |  |  |

**Table S2. Intracellular metabolites of TCA cycle detected from Control or *NbClpC1*/*C2* co-suppressed plants (ClpC) using CE-TOF-MS**

| **ID** | **HMT DB^†^ Compound name** | **m/z** | **MT** |  | | | | **Comparative analysis** | |
| --- | --- | --- | --- | --- | --- | --- | --- | --- | --- |
|  |  |  |  | **Control** | | **ClpC** | | **Control vs ClpC** | |
|  |  |  |  | **Mean** | **Standard deviation** | **mean** | **Standard deviation** | **Ratio^¶^** | ***p*-value^\|\|^** |
| A_0026 | cis-Aconitic acid | 173.009 | 30.440 | N.D. | N.A. | 0.000445 | 0.000003 | 1< | N.A. |
| A_0034 | Citric acid | 191.019 | 27.630 | 0.0180809 | 0.001779 | 0.306653 | 0.091248 | 17.0 | 0.032* |
| A_0012 | Succinic acid | 117.019 | 21.605 | 0.0003616 | 0.000027 | 0.001535 | 0.000396 | 4.2 | 0.035* |
| A_0010 | Fumaric acid | 115.003 | 27.065 | 0.0006934 | 0.000185 | 0.000416 | 0.0002 | 0.6 | 0.153 |
| A_0016 | Malic acid | 133.014 | 21.698 | 1.0380618 | 0.247251 | 0.204184 | 0.138736 | 0.2 | 0.013* |

| ID consists of analysis mode and number. 'C' and 'A' showed cation and anion modes, respectively. | | | | |
| --- | --- | --- | --- | --- |
| N.D.: Not Detected. The metabolite which was below detection limits. | |  |  |  |
| N.A.: Not Available. The calculation was not possible. |  |  |  |  |
| ^†^Putative metabolites which were assigned on the basis of m/z and MT | |  |  |  |
| ^¶^In ratio calculation, the latter was denominator. |  |  |  |  |
| ^\|\|^*p*-value in Welch's t-test.*<0.05, **<0.01, ***<0.001 |  |  |  |  |
| They were sorted by the ratio of ClpC to Control in descending order. | | |  |  |

**Table S3.** **Intracellular metabolites of glutamate, glutamine, histidine and proline metabolism detected from Control or *NbClpC1*/*C2* co-suppressed plants (ClpC) using CE-TOF-MS**

| **ID** | **HMT DB^†^ Compound name** | **m/z** | **MT** |  | | | | **Comparative analysis** | |
| --- | --- | --- | --- | --- | --- | --- | --- | --- | --- |
|  |  |  |  | **Control** | | **ClpC** | | **Control vs ClpC** | |
|  |  |  |  | **Mean** | **Standard deviation** | **mean** | **Standard deviation** | **Ratio^¶^** | ***p*-value^\|\|^** |
| C_0107 | γ-Glu-Cys | 251.069 | 12.947 | N.D. | N.A. | 0.000051 | 0.000023 | 1< | N.A. |
| A_0015 | 5-Oxoproline | 128.034 | 9.840 | 0.000219 | 0.000049 | 0.03838 | 0.006133 | 175.4 | 0.008** |
| C_0084 | Arg | 175.118 | 7.206 | 0.0005294 | 0.000046 | 0.076976 | 0.014494 | 145.4 | 0.012* |
| C_0064 | Gln | 148.079 | 10.886 | 0.0006885 | 0.000216 | 0.037769 | 0.003193 | 54.9 | 0.002** |
| C_0070 | His | 156.076 | 7.391 | 0.0009947 | 0.000257 | 0.044087 | 0.004785 | 44.3 | 0.004** |
| C_0085 | Citrulline | 176.102 | 11.216 | 0.000069 | 0.000021 | 0.000946 | 0.00011 | 13.6 | 0.004** |
| C_0001 | Urea | 61.039 | 21.400 | 0.0013057 | 0.000385 | 0.007212 | 0.00256 | 5.5 | 0.054 |
| C_0017 | 2-Aminobutyric acid | 104.070 | 9.781 | 0.0001443 | 0.000036 | 0.000484 | 0.000269 | 3.4 | 0.158 |
| C_0041 | Imidazole-4-acetic acid | 127.050 | 8.155 | 0.000023 | 0.000005 | 0.000061 | 0.000011 | 2.6 | 0.014* |
| C_0099 | Spermine | 203.222 | 4.538 | 0.0002004 | 0.000011 | 0.000478 | 0.000061 | 2.4 | 0.014* |
| C_0082 | 3-Methylhistidine | 170.092 | 7.618 | 0.000065 | 0.000038 | 0.000153 | 0.000022 | 2.3 | 0.139 |
| C_0046 | N-Acetylputrescine | 131.118 | 8.581 | 0.000038 | 0.000013 | 0.000078 | 0.000022 | 2.0 | 0.075 |
| C_0116 | Glutathione (GSSG)_divalent | 307.082 | 12.340 | 0.0003861 | 0.000078 | 0.000776 | 0.000096 | 2.0 | 0.006** |
| C_0024 | Histamine | 112.087 | 4.843 | 0.000027 | 0.000002 | 0.000053 | 0.000007 | 2.0 | 0.021* |
| C_0086 | Cys-Gly | 179.047 | 9.349 | 0.000027 | 0.000004 | 0.000049 | 0.000008 | 1.8 | 0.029* |
| C_0063 | Glu | 148.060 | 11.086 | 0.0625691 | 0.006837 | 0.09708 | 0.010315 | 1.6 | 0.012* |
| C_0050 | Ornithine | 133.096 | 6.925 | 0.0001347 | 0.000088 | 0.00019 | 0.000074 | 1.4 | 0.458 |
| C_0059 | 4-Guanidinobutyric acid | 146.093 | 8.415 | 0.000133 | 0.000016 | 0.000149 | 0.000021 | 1.1 | 0.382 |
| C_0117 | Glutathione (GSH) | 308.090 | 13.397 | 0.0108377 | 0.001288 | 0.011527 | 0.002917 | 1.1 | 0.735 |
| C_0060 | Spermidine | 146.165 | 4.597 | 0.0021632 | 0.000433 | 0.001543 | 0.00013 | 0.7 | 0.122 |
| C_0027 | Pro | 116.071 | 10.978 | 0.0556201 | 0.021324 | 0.038265 | 0.027243 | 0.7 | 0.437 |
| C_0008 | Putrescine | 89.108 | 4.775 | 0.0007049 | 0.000244 | 0.000419 | 0.000052 | 0.6 | 0.176 |
| C_0047 | Hydroxyproline | 132.066 | 12.214 | 0.0003885 | 0.000121 | 0.000081 | 0.000051 | 0.2 | 0.033* |

| ID consists of analysis mode and number. 'C' and 'A' showed cation and anion modes, respectively. | | | | |
| --- | --- | --- | --- | --- |
| N.D.: Not Detected. The metabolite which was below detection limits. | |  |  |  |
| N.A.: Not Available. The calculation was not possible. |  |  |  |  |
| ^†^Putative metabolites which were assigned on the basis of m/z and MT | |  |  |  |
| ^¶^In ratio calculation, the latter was denominator. |  |  |  |  |
| ^\|\|^*p*-value in Welch's t-test.*<0.05, **<0.01, ***<0.001 |  |  |  |  |
| In Gln where peak intensity was saturated, relative area was calculated using ^13^C data. | | | | |
| They were sorted by the ratio of ClpC to Control in descending order. | | |  |  |

**Table S4. Intracellular metabolites of glycine, serine and cysteine metabolism detected from Control or *NbClpC1*/*C2* co-suppressed plants (ClpC) using CE-TOF-MS**

| **ID** | **HMT DB^†^ Compound name** | **m/z** | **MT** |  | | | | **Comparative analysis** | |
| --- | --- | --- | --- | --- | --- | --- | --- | --- | --- |
|  |  |  |  | **Control** | | **ClpC** | | **Control vs ClpC** | |
|  |  |  |  | **Mean** | **Standard deviation** | **mean** | **Standard deviation** | **Ratio^¶^** | ***p*-value^\|\|^** |
| C_0022 | Hypotaurine | 110.027 | 18.327 | 0.0001178 | 0.00042 | 0.001236 | 0.000447 | 10.5 | 0.048* |
| C_0090 | Phosphorylcholine | 184.073 | 20.770 | 0.001488 | 0.000834 | 0.010589 | 0.00302 | 7.1 | 0.028* |
| C_0020 | Choline | 104.107 | 6.954 | 0.0043822 | 0.002807 | 0.025357 | 0.004999 | 5.8 | 0.007** |
| C_0021 | Ser | 106.050 | 10.120 | 0.0081975 | 0.00251 | 0.043741 | 0.015811 | 5.3 | 0.057 |
| C_0032 | Thr | 120.065 | 10.634 | 0.0069943 | 0.000492 | 0.031652 | 0.008462 | 4.5 | 0.037* |
| C_0003 | Gly | 76.039 | 8.454 | 0.0017427 | 0.000295 | 0.007228 | 0.003265 | 4.1 | 0.099 |
| C_0036 | Cys | 122.027 | 11.480 | 0.000184 | 0.000028 | 0.000719 | 0.000197 | 3.9 | 0.040* |
| C_0066 | Met | 150.059 | 10.825 | 0.0006122 | 0.000112 | 0.001014 | 0.000314 | 1.7 | 0.146 |
| C_0028 | Betaine | 118.086 | 11.443 | 0.0004193 | 0.000052 | 0.000593 | 0.000106 | 1.4 | 0.086 |
| A_0020 | Citramalic acid | 147.029 | 18.080 | 0.0009725 | 0.0003 | 0.0007 | 0.000237 | 0.7 | 0.288 |
| C_0118 | S-Adenosylmethionine | 399.144 | 7.208 | 0.0002602 | 0.000015 | 0.000121 | 0.000016 | 0.5 | 0.000*** |
| C_0109 | Glycerophosphocholine | 258.109 | 21.896 | 0.0066867 | 0.000466 | 0.001531 | 0.000309 | 0.2 | 0.000*** |
| A_0025 | Glycerol 3-phosphate | 171.006 | 12.563 | 0.0003606 | 0.000059 | 0.000063 | 0.000008 | 0.2 | 0.012* |
| A_0002 | Glycolic acid | 75.009 | 13.136 | 0.0015659 | 0.000767 | N.D. | N.A. | <1 | N.A. |

| ID consists of analysis mode and number. 'C' and 'A' showed cation and anion modes, respectively. | | | | |
| --- | --- | --- | --- | --- |
| N.D.: Not Detected. The metabolite which was below detection limits. | |  |  |  |
| N.A.: Not Available. The calculation was not possible. |  |  |  |  |
| ^†^Putative metabolites which were assigned on the basis of m/z and MT | |  |  |  |
| ^¶^In ratio calculation, the latter was denominator. |  |  |  |  |
| ^\|\|^*p*-value in Welch's t-test.*<0.05, **<0.01, ***<0.001 |  |  |  |  |
| They were sorted by the ratio of ClpC to Control in descending order. | | |  |  |

**Table S5. Intracellular metabolites of aspartate, alanine and lysine metabolism detected from Control or *NbClpC1*/*C2* co-suppressed plants (ClpC) using CE-TOF-MS**

| **ID** | **HMT DB^†^ Compound name** | **m/z** | **MT** |  | | | | **Comparative analysis** | |
| --- | --- | --- | --- | --- | --- | --- | --- | --- | --- |
|  |  |  |  | **Control** | | **ClpC** | | **Control vs ClpC** | |
|  |  |  |  | **Mean** | **Standard deviation** | **mean** | **Standard deviation** | **Ratio^¶^** | ***p*-value^\|\|^** |
| C_0113 | Saccharopine | 277.139 | 10.931 | N.D. | N.A. | 0.001109 | 0.000687 | 1< | N.A. |
| C_0075 | 2-Aminoadipic acid | 162.075 | 11.086 | 0.000068 | 0.000011 | 0.004973 | 0.002308 | 72.8 | 0.067 |
| C_0053 | Asn | 134.066 | 10.598 | 0.0001703 | 0.000099 | 0.011987 | 0.001024 | 70.4 | 0.002** |
| C_0061 | Lys | 147.112 | 6.976 | 0.0015157 | 0.000234 | 0.079861 | 0.020932 | 52.7 | 0.023* |
| C_0094 | N6,N6,N6-Trimethyllysine | 189.159 | 7.291 | 0.000057 | 0.000017 | 0.001834 | 0.000331 | 32.1 | 0.011* |
| C_0009 | Ala | 90.055 | 9.159 | 0.0077749 | 0.001197 | 0.049817 | 0.01258 | 6.4 | 0.028* |
| C_0043 | Pipecolic acid | 130.086 | 10.387 | 0.0002764 | 0.000122 | 0.001759 | 0.000544 | 6.4 | 0.037* |
| C_0052 | Asp | 134.044 | 11.732 | 0.0256789 | 0.006211 | 0.125632 | 0.018243 | 4.9 | 0.006** |
| C_0010 | β-Ala | 90.055 | 7.471 | 0.0004027 | 0.000133 | 0.000922 | 0.000063 | 2.3 | 0.010* |
| A_0008 | 3-Hydroxybutyric acid | 103.040 | 9.838 | 0.0001567 | 0.000014 | N.D. | N.A. | <1 | N.A. |

| ID consists of analysis mode and number. 'C' and 'A' showed cation and anion modes, respectively. | | | | |
| --- | --- | --- | --- | --- |
| N.D.: Not Detected. The metabolite which was below detection limits. | |  |  |  |
| N.A.: Not Available. The calculation was not possible. |  |  |  |  |
| ^†^Putative metabolites which were assigned on the basis of m/z and MT | |  |  |  |
| ^¶^In ratio calculation, the latter was denominator. |  |  |  |  |
| ^\|\|^*p*-value in Welch's t-test.*<0.05, **<0.01, ***<0.001 |  |  |  |  |
| In Asn where peak intensity was saturated, relative area was calculated using ^13^C data. | | | | |
| They were sorted by the ratio of ClpC to Control in descending order. | | |  |  |

**Table S6.** **Intracellular metabolites of branched chain amino acid metabolism detected from Control or *NbClpC1*/*C2* co-suppressed plants (ClpC) using CE-TOF-MS**

| **ID** | **HMT DB^†^ Compound name** | **m/z** | **MT** |  | | | | **Comparative analysis** | |
| --- | --- | --- | --- | --- | --- | --- | --- | --- | --- |
|  |  |  |  | **Control** | | **ClpC** | | **Control vs ClpC** | |
|  |  |  |  | **Mean** | **Standard deviation** | **mean** | **Standard deviation** | **Ratio^¶^** | ***p*-value^\|\|^** |
| C_0051 | Leu | 133.105 | 10.411 | 0.000336 | 0.000053 | 0.014673 | 0.002021 | 43.7 | 0.007** |
| C_0030 | Val | 118.086 | 10.133 | 0.0071986 | 0.0016 | 0.146696 | 0.030952 | 20.4 | 0.016* |
| C_0049 | Ile | 132.101 | 10.334 | 0.0059692 | 0.000627 | 0.062732 | 0.008139 | 10.5 | 0.007** |
| C_0016 | 3-Aminoisobutyric acid | 104.070 | 7.959 | 0.000064 | 0.000017 | 0.000486 | 0.000014 | 7.5 | 0.000*** |
| A_0004 | 3-Hydroxypropionic acid | 89.025 | 10.832 | 0.000146 | 0.000018 | N.D. | N.A. | <1 | N.A. |

| ID consists of analysis mode and number. 'C' and 'A' showed cation and anion modes, respectively. | | | | |
| --- | --- | --- | --- | --- |
| N.D.: Not Detected. The metabolite which was below detection limits. | |  |  |  |
| N.A.: Not Available. The calculation was not possible. |  |  |  |  |
| ^†^Putative metabolites which were assigned on the basis of m/z and MT | |  |  |  |
| ^¶^In ratio calculation, the latter was denominator. |  |  |  |  |
| ^\|\|^*p*-value in Welch's t-test.*<0.05, **<0.01, ***<0.001 |  |  |  |  |
| In Leu where peak intensity was saturated, relative area was calculated using ^13^C data. | | | | |
| They were sorted by the ratio of ClpC to Control in descending order. | | |  |  |

**Table S7.** **Intracellular metabolites of aromatic amino acid metabolism detected from Control or *NbClpC1*/*C2* co-suppressed plants (ClpC) using CE-TOF-MS**

| **ID** | **HMT DB^†^ Compound name** | **m/z** | **MT** |  | | | | **Comparative analysis** | |
| --- | --- | --- | --- | --- | --- | --- | --- | --- | --- |
|  |  |  |  | **Control** | | **ClpC** | | **Control vs ClpC** | |
|  |  |  |  | **Mean** | **Standard deviation** | **mean** | **Standard deviation** | **Ratio^¶^** | ***p*-value^\|\|^** |
| C_0069 | Dopamine | 154.087 | 8.826 | N.D. | N.A. | 0.000034 | 0.000005 | 1< | N.A. |
| C_0089 | Tyr | 182.080 | 11.516 | 0.0008257 | 0.000287 | 0.042575 | 0.008268 | 51.6 | 0.013* |
| C_0081 | 3-Methoxytyramine | 168.101 | 8.977 | 0.000016 | 0.000002 | 0.000298 | 0.000146 | 18.1 | 0.079 |
| C_0080 | Phe | 166.086 | 11.251 | 0.0020845 | 0.000201 | 0.028049 | 0.012886 | 13.5 | 0.073 |
| C_0100 | Trp | 205.096 | 11.119 | 0.0011684 | 0.000748 | 0.005723 | 0.001081 | 4.9 | 0.006** |
| C_0101 | Kynurenine | 209.091 | 10.050 | 0.000021 | 0.000009 | 0.000041 | 0.000003 | 2.0 | 0.196 |
| C_0058 | Tyramine | 138.091 | 8.405 | 0.0026671 | 0.000678 | 0.003784 | 0.000283 | 1.4 | 0.088 |
| C_0038 | 2-Phenylethylamine | 122.096 | 7.850 | 0.0198267 | 0.003413 | 0.016458 | 0.003948 | 0.8 | 0.327 |
| C_0057 | Anthranilic acid | 138.055 | 10.613 | 0.0001873 | 0.00021 | N.D. | N.A. | <1 | N.A. |
| C_0073 | Tryptamine | 161.107 | 8.446 | 0.000059 | 0.000019 | N.D. | N.A. | <1 | N.A. |

| ID consists of analysis mode and number. 'C' and 'A' showed cation and anion modes, respectively. | | | | |
| --- | --- | --- | --- | --- |
| N.D.: Not Detected. The metabolite which was below detection limits. | |  |  |  |
| N.A.: Not Available. The calculation was not possible. |  |  |  |  |
| ^†^Putative metabolites which were assigned on the basis of m/z and MT | |  |  |  |
| ^¶^In ratio calculation, the latter was denominator. |  |  |  |  |
| ^\|\|^*p*-value in Welch's t-test.*<0.05, **<0.01, ***<0.001 |  |  |  |  |
| They were sorted by the ratio of ClpC to Control in descending order. | | |  |  |

**Table S8.** **Intracellular metabolites of purine and pyrimidine metabolism detected from Control or *NbClpC1*/*C2* co-suppressed plants (ClpC) using CE-TOF-MS**

| **ID** | **HMT DB^†^ Compound name** | **m/z** | **MT** |  | | | | **Comparative analysis** | |
| --- | --- | --- | --- | --- | --- | --- | --- | --- | --- |
|  |  |  |  | **Control** | | **ClpC** | | **Control vs ClpC** | |
|  |  |  |  | **Mean** | **Standard deviation** | **mean** | **Standard deviation** | **Ratio^¶^** | ***p*-value^\|\|^** |
| A_0073 | UTP | 482.960 | 13.077 | N.D. | N.A. | 0.000107 | 0.000025 | 1< | N.A. |
| A_0075 | ATP | 505.986 | 12.210 | N.D. | N.A. | 0.000123 | 0.000035 | 1< | N.A. |
| C_0068 | Guanine | 152.056 | 8.424 | N.D. | N.A. | 0.000488 | 0.000201 | 1< | N.A. |
| C_0105 | Cytidine | 244.092 | 9.804 | N.D. | N.A. | 0.000122 | 0.000037 | 1< | N.A. |
| C_0106 | Uridine | 245.078 | 22.346 | N.D. | N.A. | 0.00018 | 0.000031 | 1< | N.A. |
| C_0115 | Guanosine | 284.098 | 12.719 | 0.000052 | 0.000007 | 0.000196 | 0.000083 | 3.7 | 0.095 |
| C_0111 | Adenosine | 268.103 | 10.009 | 0.000024 | 0.000003 | 0.000063 | 0.000018 | 2.5 | 0.064 |
| A_0064 | UDP | 402.993 | 12.076 | 0.000045 | N.A. | 0.000076 | 0.000026 | 1.7 | N.A. |
| A_0068 | ADP | 426.020 | 11.240 | 0.000096 | 0.000018 | 0.000084 | 0.000027 | 0.9 | 0.585 |

| ID consists of analysis mode and number. 'C' and 'A' showed cation and anion modes, respectively. | | | | |
| --- | --- | --- | --- | --- |
| N.D.: Not Detected. The metabolite which was below detection limits. | |  |  |  |
| N.A.: Not Available. The calculation was not possible. |  |  |  |  |
| ^†^Putative metabolites which were assigned on the basis of m/z and MT | |  |  |  |
| ^¶^In ratio calculation, the latter was denominator. |  |  |  |  |
| ^\|\|^*p*-value in Welch's t-test.*<0.05, **<0.01, ***<0.001 |  |  |  |  |
| They were sorted by the ratio of ClpC to Control in descending order. | | |  |  |

**Table S9.** **Intracellular metabolites detected from Control or *NbClpC1*/*C2* co-suppressed plants (ClpC) using CE-TOF-MS which were not categorized on the basis of pathway metabolism**

| **ID** | **HMT DB^†^ Compound name** | **m/z** | **MT** |  | | | | **Comparative analysis** | |
| --- | --- | --- | --- | --- | --- | --- | --- | --- | --- |
|  |  |  |  | **Control** | | **ClpC** | | **Control vs ClpC** | |
|  |  |  |  | **Mean** | **Standard deviation** | **mean** | **Standard deviation** | **Ratio^¶^** | ***p*-value^\|\|^** |
| A_0018 | Ethanolamine phosphate | 140.011 | 8.254 | N.D. | N.A. | 0.000184 | 0.000019 | 1< | N.A. |
| A_0021 | Phthalic acid | 165.019 | 16.060 | N.D. | N.A. | 0.00015 | N.A. | 1< | N.A. |
| A_0029 | 2-Isopropylmalic acid | 175.060 | 14.150 | N.D. | N.A. | 0.000617 | 0.000235 | 1< | N.A. |
| A_0043 | Biotin | 243.082 | 7.896 | N.D. | N.A. | 0.000096 | 0.000025 | 1< | N.A. |
| A_0044 | Glucosamine 6-phosphate | 258.037 | 8.507 | N.D. | N.A. | 0.000416 | 0.00019 | 1< | N.A. |
| C_0014 | Homoserinelactone | 102.055 | 7.138 | N.D. | N.A. | 0.000048 | 0.000014 | 1< | N.A. |
| C_0031 | 2,4-Diaminobutyric acid | 119.081 | 6.957 | N.D. | N.A. | 0.000174 | 0.000025 | 1< | N.A. |
| C_0045 | trans-Glutaconic acid | 131.032 | 23.135 | N.D. | N.A. | 0.000066 | N.A. | 1< | N.A. |
| C_0065 | Nornicotine | 149.106 | 5.159 | N.D. | N.A. | 0.012213 | 0.001653 | 1< | N.A. |
| C_0071 | Allantoin | 159.050 | 22.287 | N.D. | N.A. | 0.016696 | 0.001296 | 1< | N.A. |
| C_0074 | N6-Methyllysine | 161.128 | 7.156 | N.D. | N.A. | 0.000225 | 0.000045 | 1< | N.A. |
| C_0087 | S-Carboxymethylcysteine | 180.032 | 13.206 | N.D. | N.A. | 0.000166 | 0.000044 | 1< | N.A. |
| C_0091 | Gly-Leu | 189.123 | 9.904 | N.D. | N.A. | 0.000086 | 0.000022 | 1< | N.A. |
| C_0092 | N6-Acetyllysine | 189.123 | 11.582 | N.D. | N.A. | 0.000342 | 0.000103 | 1< | N.A. |
| C_0093 | Nω-Methylarginine | 189.134 | 7.565 | N.D. | N.A. | 0.00127 | 0.000246 | 1< | N.A. |
| C_0097 | ADMA | 203.149 | 7.775 | N.D. | N.A. | 0.00072 | 0.000049 | 1< | N.A. |
| C_0098 | SDMA | 203.149 | 7.897 | N.D. | N.A. | 0.000178 | 0.000017 | 1< | N.A. |
| C_0110 | Thiamine | 265.111 | 6.691 | N.D. | N.A. | 0.000125 | 0.000035 | 1< | N.A. |
| C_0078 | S-Methylmethionine | 164.073 | 7.398 | 0.0002754 | 0.000044 | 0.023549 | 0.004685 | 85.5 | 0.013* |
| C_0039 | Nicotinic acid | 124.039 | 10.168 | 0.000027 | N.A. | 0.000401 | 0.000184 | 14.8 | N.A. |
| C_0018 | GABA | 104.070 | 7.800 | 0.001047 | 0.000286 | 0.010309 | 0.003535 | 9.8 | 0.044* |
| C_0056 | p-Aminobenzoic acid | 138.055 | 9.884 | 0.000023 | 0.000009 | 0.000189 | 0.000041 | 8.1 | 0.014* |
| C_0002 | Ethanolamine | 62.061 | 6.460 | 0.0001613 | 0.000038 | 0.000853 | 0.000131 | 5.3 | 0.008** |
| C_0079 | Methionine sulfoxide | 166.052 | 11.968 | 0.0001042 | 0.000015 | 0.000438 | 0.000078 | 4.2 | 0.015* |
| C_0029 | 5-Aminovaleric acid | 118.086 | 8.154 | 0.0006822 | 0.000391 | 0.002434 | 0.000374 | 3.6 | 0.005** |
| C_0083 | N5-Ethylglutamine | 175.107 | 11.530 | 0.0001323 | 0.000021 | 0.000426 | 0.000048 | 3.2 | 0.003** |
| C_0119 | Cysteine glutathione disulfide | 427.095 | 11.825 | 0.000024 | 0.000002 | 0.000054 | 0.000017 | 2.2 | 0.093 |
| C_0072 | Ala-Ala | 161.091 | 9.393 | 0.000094 | 0.000043 | 0.000204 | 0.000056 | 2.2 | 0.061 |

*(Continued)*

| **ID** | **HMT DB^†^ Compound name** | **m/z** | **MT** |  | | | | **Comparative analysis** | |
| --- | --- | --- | --- | --- | --- | --- | --- | --- | --- |
|  |  |  |  | **Control** | | **ClpC** | | **Control vs ClpC** | |
|  |  |  |  | **Mean** | **Standard deviation** | **mean** | **Standard deviation** | **Ratio^¶^** | ***p*-value^\|\|^** |
| C_0114 | 1-Methyladenosine | 282.119 | 10.083 | 0.000054 | 0.000005 | 0.000113 | 0.000016 | 2.1 | 0.016* |
| C_0096 | Gly-Asp | 191.065 | 10.132 | 0.000058 | 0.000011 | 0.000109 | 0.000020 | 1.9 | 0.032* |
| C_0067 | 3-Methyladenine | 150.077 | 8.048 | 0.0002704 | 0.000083 | 0.000496 | 0.000104 | 1.8 | 0.045* |
| C_0062 | O-Acetylserine | 148.060 | 12.802 | 0.0001691 | 0.000008 | 0.000308 | 0.000078 | 1.8 | 0.090 |
| C_0023 | Cytosine | 112.051 | 7.356 | 0.000012 | 0.000002 | 0.000021 | 0.000023 | 1.7 | 0.011* |
| C_0037 | 2-Amino-2-(hydroxymethyl)-1,3-propanediol | 122.080 | 8.431 | 0.000052 | 0.000022 | 0.000078 | 0.000017 | 1.5 | 0.194 |
| A_0039 | 6,8-Thioctic acid | 205.035 | 8.666 | 0.000048 | 0.000009 | 0.000070 | 0.000013 | 1.5 | 0.126 |
| C_0005 | Pyridine | 80.049 | 5.990 | 0.0003372 | 0.000097 | 0.000468 | 0.000123 | 1.4 | 0.225 |
| C_0077 | Nicotine | 163.122 | 5.367 | 0.023549 | 0.005349 | 0.032273 | 0.009866 | 1.4 | 0.269 |
| A_0014 | Benzoic acid | 121.030 | 10.175 | 0.000071 | 0.000009 | 0.000092 | 0.000009 | 1.3 | 0.117 |
| C_0012 | 1-Methyl-2-pyrrolidone | 100.075 | 22.142 | 0.0002061 | 0.000028 | 0.000239 | 0.000009 | 1.2 | 0.175 |
| C_0013 | Cyclohexylamine | 100.112 | 7.798 | 0.000095 | 0.000033 | 0.000107 | 0.000019 | 1.1 | 0.656 |
| C_0042 | Quinoline | 130.066 | 7.302 | 0.000035 | 0.000001 | 0.000038 | 0.000005 | 1.1 | 0.439 |
| A_0077 | UDP-glucose UDP-galactose | 565.047 | 8.919 | 0.000424 | 0.000084 | 0.000369 | 0.000077 | 0.9 | 0.456 |
| A_0078 | NAD+ | 662.102 | 6.753 | 0.000061 | 0.000008 | 0.000052 | 0.000010 | 0.9 | 0.326 |
| C_0044 | Octylamine | 130.158 | 8.574 | 70.000078 | 0.000026 | 0.000060 | 0.000011 | 0.8 | 0.359 |
| C_0095 | Castanospermine | 190.107 | 9.124 | 0.000066 | 0.000026 | 0.000045 | 0.000008 | 0.7 | 0.470 |
| A_0006 | Malonic acid | 103.003 | 34.499 | 0.0025821 | 0.000539 | 0.001588 | 0.000208 | 0.6 | 0.070 |
| C_0076 | Anabasine | 163.122 | 5.555 | 0.0080292 | 0.00164 | 0.004828 | 0.000539 | 0.6 | 0.065 |
| A_0048 | myo-Inositol 1-phosphate myo-Inositol 3-phosphate | 259.021 | 10.601 | 0.0002141 | 0.000049 | 0.000104 | 0.000033 | 0.5 | 0.040* |
| C_0102 | N-Acetylglucosamine | 222.097 | 22.320 | 0.0007631 | 0.000593 | 0.000349 | 0.000097 | 0.5 | 0.349 |
| A_0037 | Gluconic acid | 195.050 | 8.390 | 0.0024886 | 0.000341 | 0.001095 | 0.000202 | 0.4 | 0.007** |
| C_0006 | Piperidine | 86.097 | 7.056 | 0.0001214 | 0.000022 | 0.000053 | 0.000016 | 0.4 | 0.017* |
| A_0045 | Glucose 1-phosphate | 259.021 | 10.464 | 0.0002797 | 0.000020 | 0.000118 | 0.000041 | 0.4 | 0.009** |
| C_0108 | Dyphylline | 255.107 | 22.346 | 0.0004867 | 0.000068 | 0.000193 | 0.000031 | 0.4 | 0.009** |
| C_0004 | Isopropanolamine | 76.075 | 7.108 | 0.000057 | 0.000013 | 0.000019 | 0.000002 | 0.3 | 0.037* |
| A_0066 | Trehalose 6-phosphate | 421.079 | 8.771 | 0.0002636 | 0.000049 | 0.000065 | 0.000013 | 0.2 | 0.015* |
| A_0040 | Ethyl glucuronide | 221.066 | 7.746 | 0.0005006 | 0.000261 | 0.000098 | N.A. | 0.2 | N.A. |
| C_0015 | Hexylamine | 102.127 | 7.902 | 0.0001793 | 0.000042 | 0.000033 | 0.000011 | 0.2 | 0.018* |
| A_0036 | Galacturonic acid | 193.033 | 8.603 | 0.0003693 | 0.000009 | 0.000067 | 0.000022 | 0.2 | 0.000*** |

*(Continued)*

| **ID** | **HMT DB^†^ Compound name** | **m/z** | **MT** |  | | | | **Comparative analysis** | |
| --- | --- | --- | --- | --- | --- | --- | --- | --- | --- |
|  |  |  |  | **Control** | | **ClpC** | | **Control vs ClpC** | |
|  |  |  |  | **Mean** | **Standard deviation** | **mean** | **Standard deviation** | **Ratio^¶^** | ***p*-value^\|\|^** |
| A_0030 | N-Formylmethionine | 176.040 | 8.289 | 0.2241575 | 0.009903 | 0.02858 | 0.006372 | 0.1 | 0.000*** |
| C_0088 | Theobromine | 181.072 | 22.307 | 0.0067163 | 0.001028 | 0.000676 | 0.000034 | 0.1 | 0.009** |
| A_0027 | Shikimic acid | 173.044 | 8.458 | 0.0010289 | 0.000075 | 0.000084 | 0.000016 | 0.1 | 0.001** |
| A_0009 | Glyceric acid | 105.019 | 10.746 | 0.0155474 | 0.006989 | 0.000747 | 0.000563 | 0.0 | 0.066 |
| A_0028 | Ascorbic acid | 175.024 | 8.784 | 0.0277005 | 0.007734 | 0.000911 | 0.000367 | 0.0 | 0.026* |
| A_0017 | Threonic acid | 135.029 | 9.628 | 0.1029809 | 0.03361 | 0.003258 | 0.002641 | 0.0 | 0.035* |
| A_0035 | Quinic acid | 191.055 | 8.591 | 0.3002112 | 0.021775 | 0.001199 | 0.000826 | 0.0 | 0.002** |
| A_0013 | 2-Hydroxyvaleric acid | 117.055 | 9.292 | 0.0002017 | 0.000078 | N.D. | N.A. | <1 | N.A. |
| C_0033 | 2-Methylserine | 120.065 | 10.520 | 0.000038 | 0.000011 | N.D. | N.A. | <1 | N.A. |
| A_0018 | Ethanolamine phosphate | 140.011 | 8.254 | N.D. | N.A. | 0.000184 | 0.000019 | 1< | N.A. |

| ID consists of analysis mode and number. 'C' and 'A' showed cation and anion modes, respectively. | | | | |
| --- | --- | --- | --- | --- |
| N.D.: Not Detected. The metabolite which was below detection limits. | |  |  |  |
| N.A.: Not Available. The calculation was not possible. |  |  |  |  |
| ^†^Putative metabolites which were assigned on the basis of m/z and MT | |  |  |  |
| ^¶^In ratio calculation, the latter was denominator. |  |  |  |  |
| ^\|\|^*p*-value in Welch's t-test.*<0.05, **<0.01, ***<0.001 |  |  |  |  |
| They were sorted by the ratio of ClpC to Control in descending order. | | |  |  |
