# Supplementary material for: Co-Suppression of NbClpC1 and NbClpC2, Encoding Clp Protease Chaperons, Elicits Significant Changes in the Metabolic Profile of Nicotiana benthamiana
Source: Plants (Basel). 2020 Feb 18;9(2):259. doi: 10.3390/plants9020259 (PMC7076384; doi:10.3390/plants9020259)
Supplement: Supplementary file 1 [file plants-09-00259-s001.zip › Supplementary_data_02-10-20.pdf]

Supplementary Materials

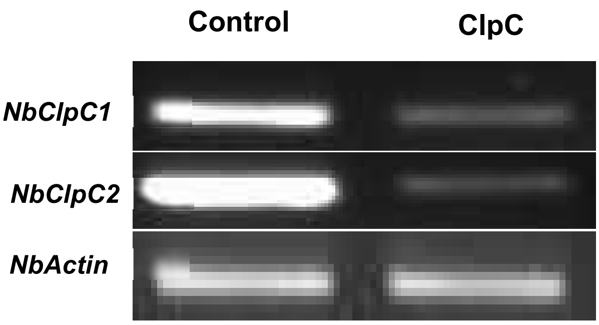

Figure S1. *NbClpC1/C2* co-suppression in *N. benthamiana*. Semi-quantitative RT-PCR analysis for identification of the silencing of *NbClpC1* and *NbClpC2* expression separately. The actin gene was used to identify the equal use of total RNA.

MATERIALS AND METHODS

Materials

Nicotiana benthamiana sample was sent from Yeungnam University to HMT under freezing condition.

Sample information are shown in Table 1.

Name	Amount (mg)	Group
NBC-1	50	
NBC-2	50	Control
NBC-3	50	
NBT-1	50	
NBT-2	50	Treatment
NBT-3	50	

Sample Preparation

After receipt of samples, 50 µL of Milli-Q water was added to the extract from *Nicotiana benthamiana*. They were subject to CE-TOFMS analysis.

CE-TOFMS ANALYSIS

Metabolome analysis (Cation and Anion Mode) was performed in the following conditions. Judging from peak shapes and intensities, they were diluted by 20% for CE-TOFMS analysis in Anion mode.

Cationic Metabolites (Cation Mode)

Device

Agilent CE-TOFMS system（Agilent Technologies, Inc）Machine No. 3

Capillary: Fused silica capillary i.d. 50 μm × 80 cm

Analytical Condition

Run buffer: Cation Buffer Solution (p/n: H3301-1001)

Rinse buffer: Cation Buffer Solution (p/n: H3301-1001)

Sample injection: Pressure injection 50 mbar, 10 sec

CE voltage: Positive, 27 kV

MS ionization: ESI Positive

MS capillary voltage: 4,000 V

MS scan range: *m/z* 50-1,000

Sheath liquid: HMT Sheath Liquid (p/n: H3301-1020)

Anionic Metabolites (Anion Mode)

Device

Agilent CE-TOFMS system（Agilent Technologies, Inc）Machine No. 5

Capillary: Fused silica capillary i.d. 50 μm × 80 cm

Analytical Condition

Run buffer: Anion Buffer Solution (p/n : H3302-1021)

Rinse buffer: Anion Buffer Solution (p/n : H3302-1022)

Sample injection: Pressure injection 50 mbar, 25 sec

CE voltage: Positive, 30 kV

MS ionization: ESI Negative

MS capillary voltage: 3,500 V

MS scan range: *m/z* 50-1,000

Sheath liquid: HMT Sheath Liquid (p/n: H3301-1020)

DATA PROCESSING AND ANALYSIS

Data Processing

Peaks detected in CE-TOFMS analysis were extracted using the automatic integration software (MasterHands ver. 2.9.0.9, Keio University-developed software). Peak information including *m/z*, migration time (MT) and area was obtained. Peak area was converted into relative peak area according to the following equation. Each peak was aligned according to similar migration time on CE and *m/z* value determined by TOFMS.

$$\text{Relative peak Area} = \frac{\text{Metabolite Peak Area}}{\text{Internal Standard Peak Area} \times \text{Sample Amount}}$$

Putative Metabolites

Putative metabolites were assigned from HMT metabolite database on the basis of *m/z* and MT. The tolerance was ±0.5 min in MT and ±10 ppm in *m/z*.

$$\text{Mass error (ppm)} = \frac{\text{Measured Value} - \text{Theoretical Value}}{\text{Measured Value}} \times 10^{6}$$

Statistical Analysis (PCA, HCA)

Principal component analysis (PCA) was performed with SampleStat ver. 3.14 (in-house software). Hierarchical cluster analysis (HCA) was done using PeakStat ver. 3.18 (in-house software).

Plotting on Pathway Map

Detected metabolites were plotted on the map (Figure 1 to 9) using VANTED (Visualization and Analysis of Networks containing Experimental Data). The pathway map in VANTED was made on the basis of metabolic pathway in human.

Quantitative Estimation of HMT Standard Metabolites

Quantitative estimation was performed in 108 metabolites including intermediates in glycolysis, TCA cycle, amino acids and nucleic acid. Concentrations of the metabolites were calculated by the normalization with peak area of internal standard. Standard curve for each metabolite was obtained by single-point at 100 μM standard metabolites.

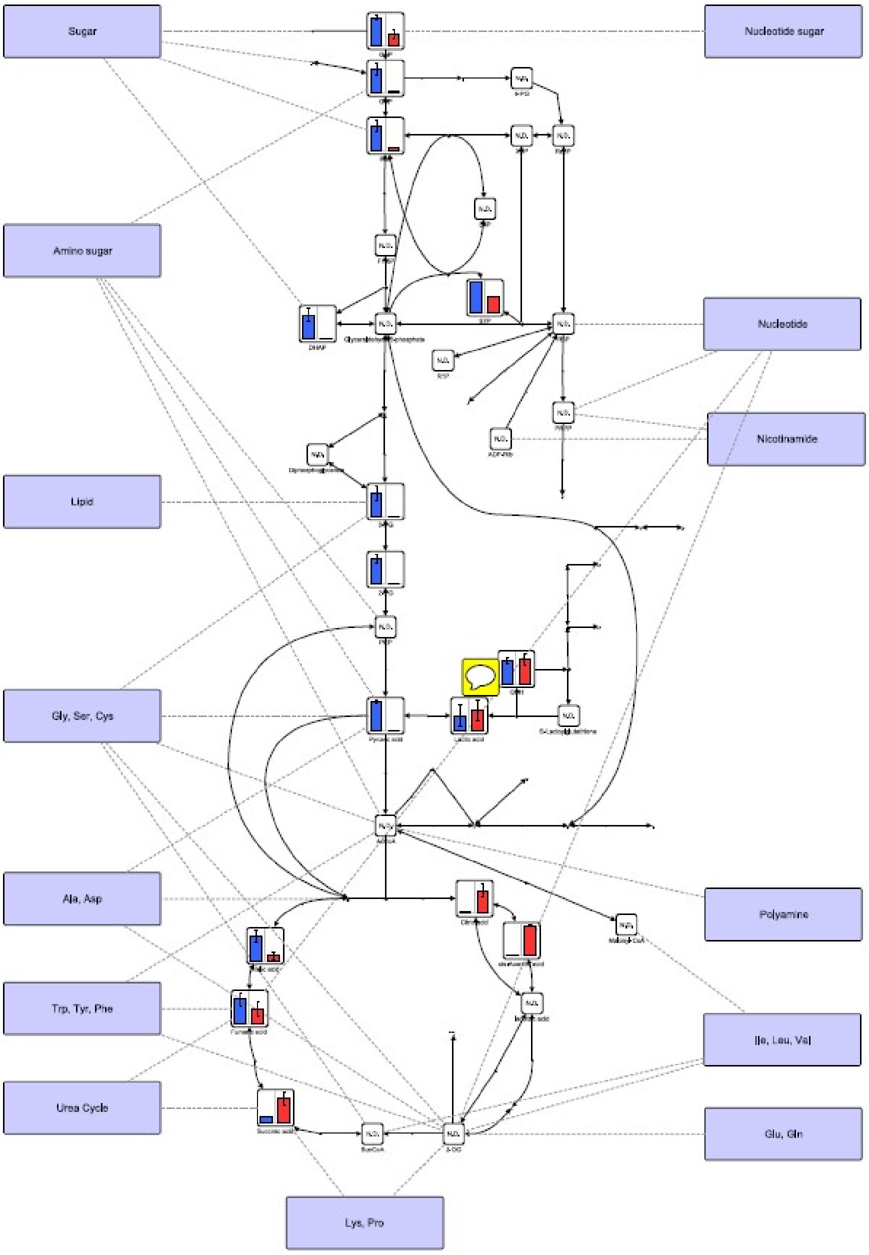

Figure S2. Pathway Map (Glycolysis, Pentose Phosphate Pathway, TCA Cycle) In HMT Standard Metabolites, detected metabolites in this study are plotted on the pathway map. Blue and Red vertical bars show Control and Treatment, respectively. N.D.: Not Detected.

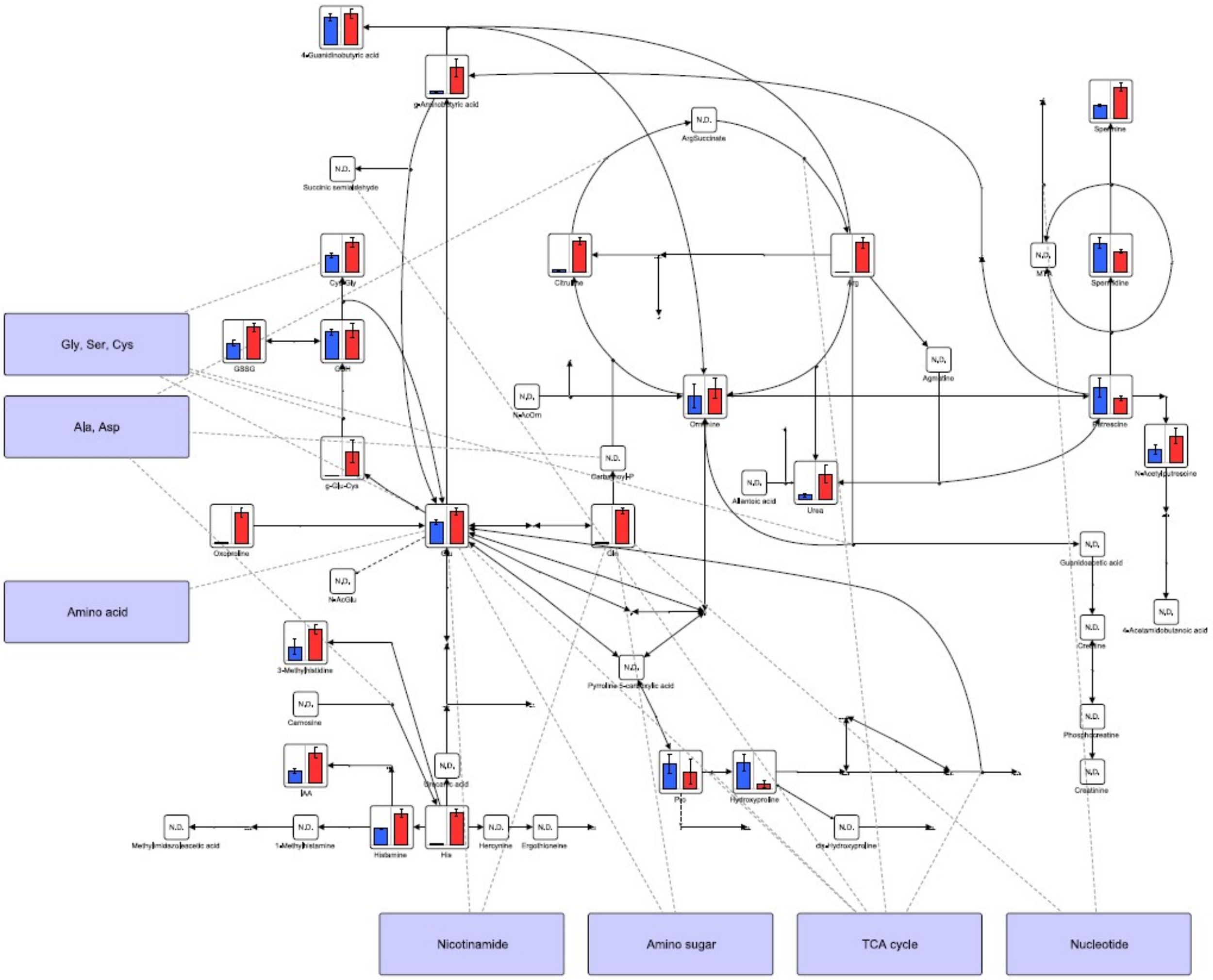

Figure S3. Pathway Map (Urea Cycle, Glu, Gln, His, Pro) In HMT Standard Metabolites, detected metabolites in this study are plotted on the pathway map. Blue and Red vertical bars show Control and Treatment, respectively. N.D.: Not Detected.

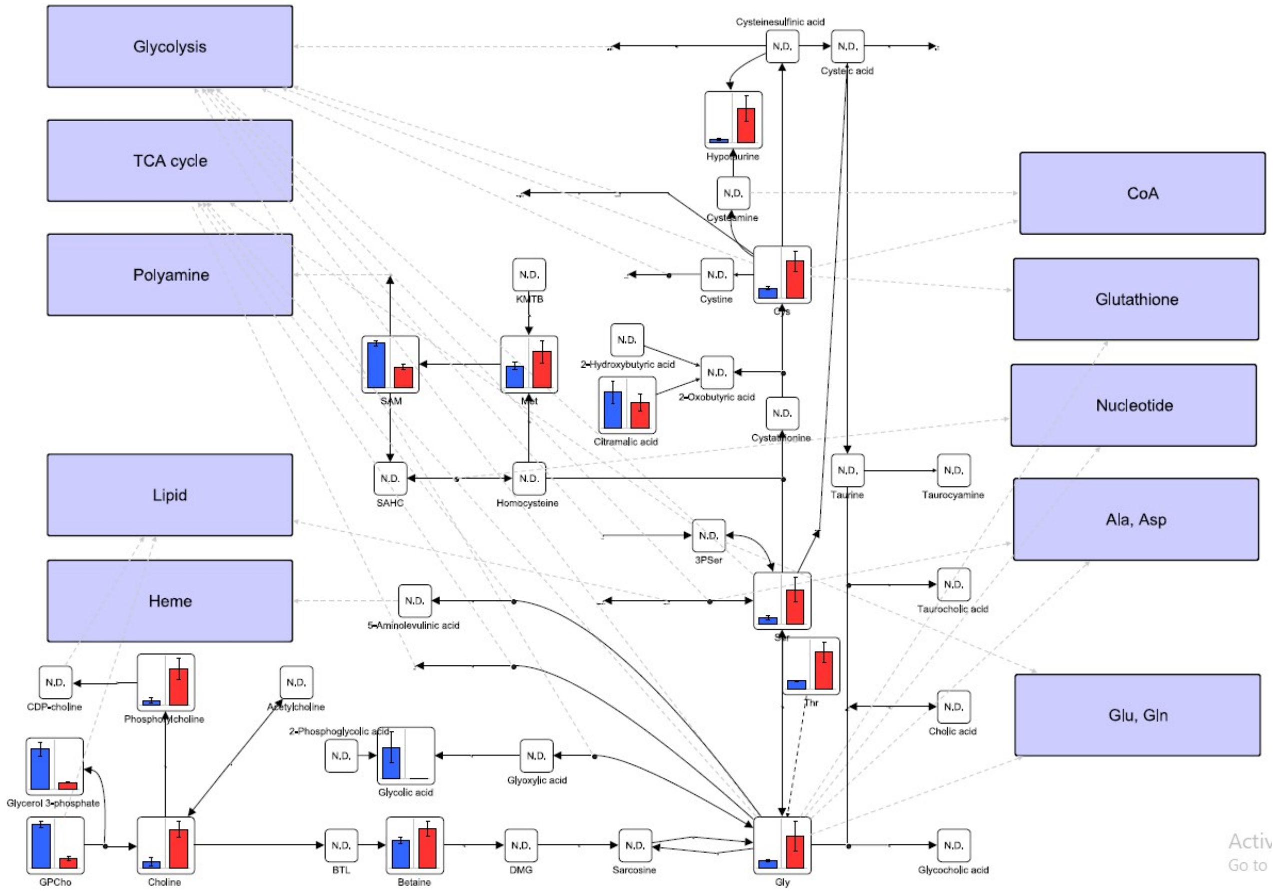

Figure S4. Pathway Map (Gly, Ser, Cys) In HMT Standard Metabolites, detected metabolites in this study are plotted on the pathway map. Blue and Red vertical bars show Control and Treatment, respectively. N.D.: Not Detected.

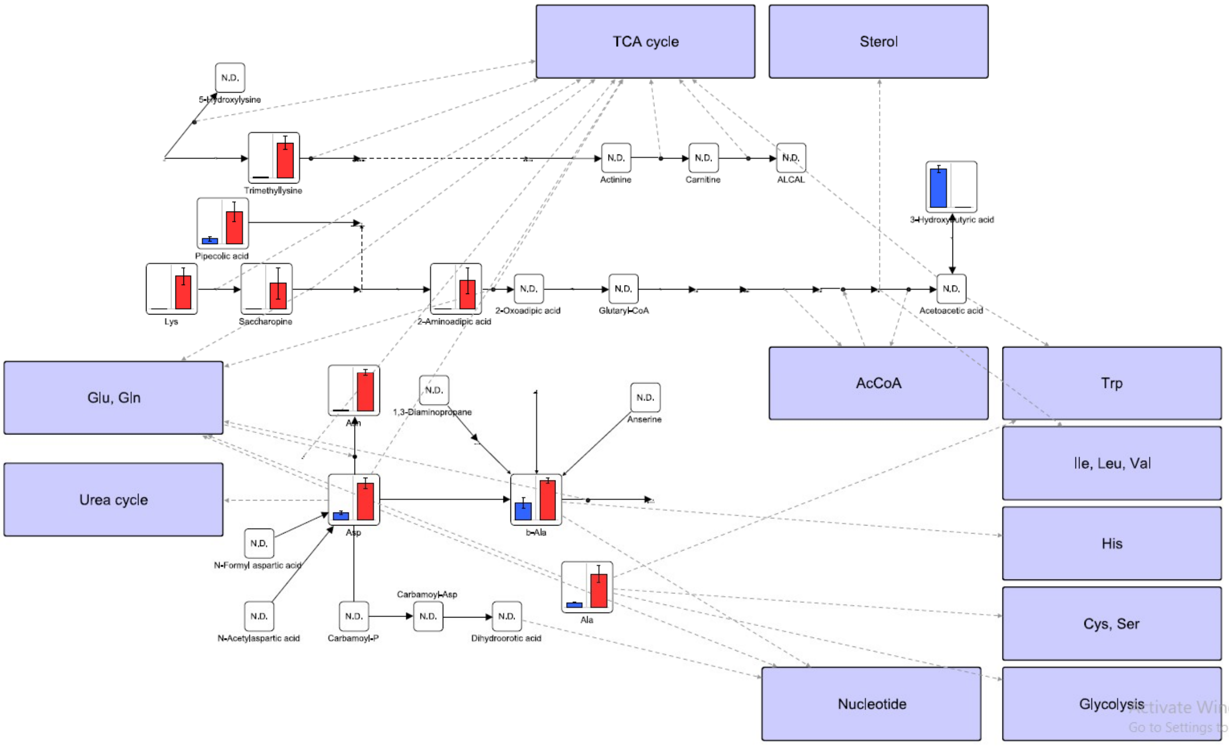

Figure S5. Pathway Map (Asp, Ala, Lys) In HMT Standard Metabolites, detected metabolites in this study are plotted on the pathway map. Blue and Red vertical bars show Control and Treatment, respectively. N.D.: Not Detected.

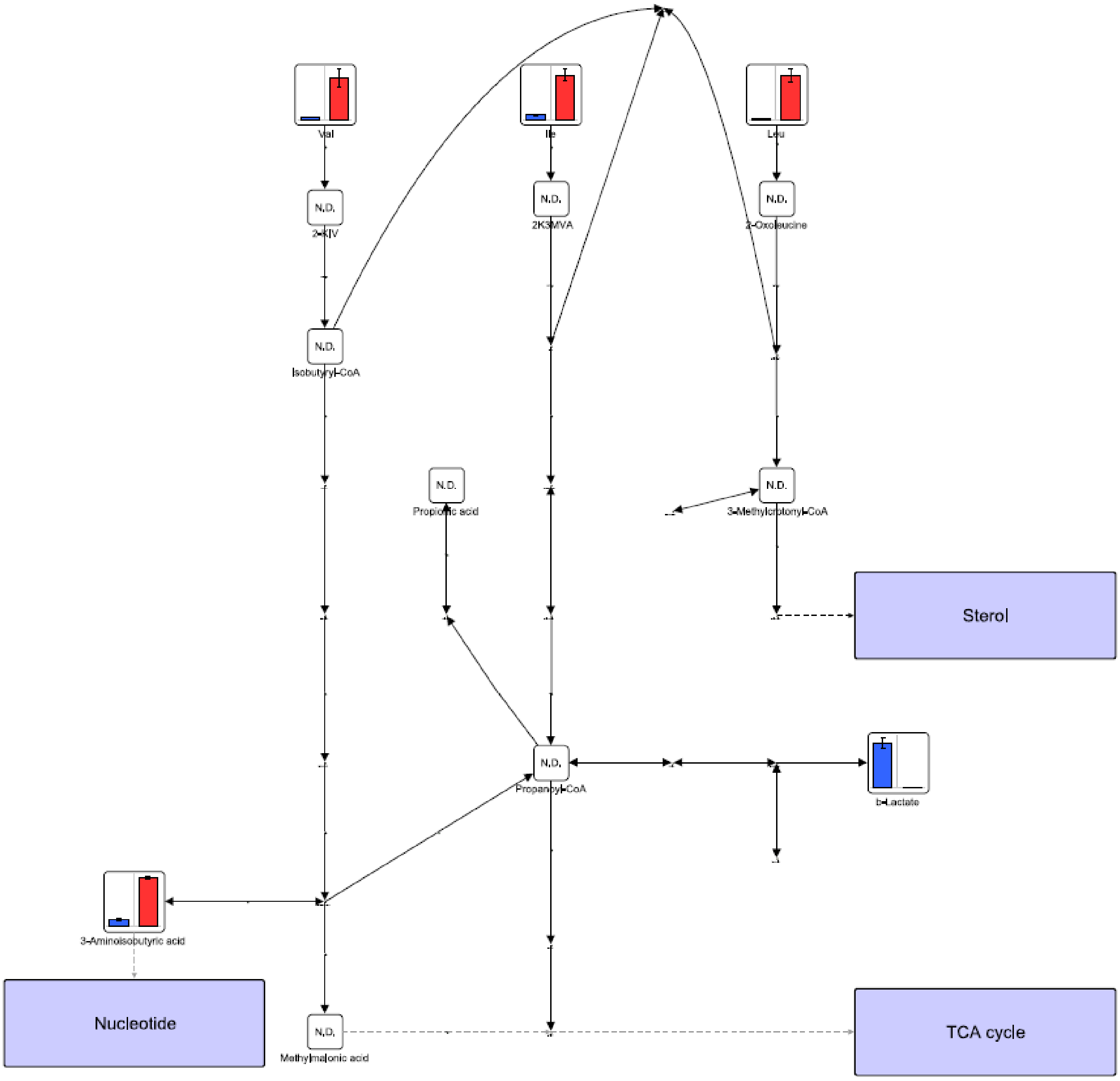

Figure S6. Pathway Map (Branched-Chain Amino Acids) In HMT Standard Metabolites, detected metabolites in this study are plotted on the pathway map. Blue and Red vertical bars show Control and Treatment, respectively. N.D.: Not Detected.

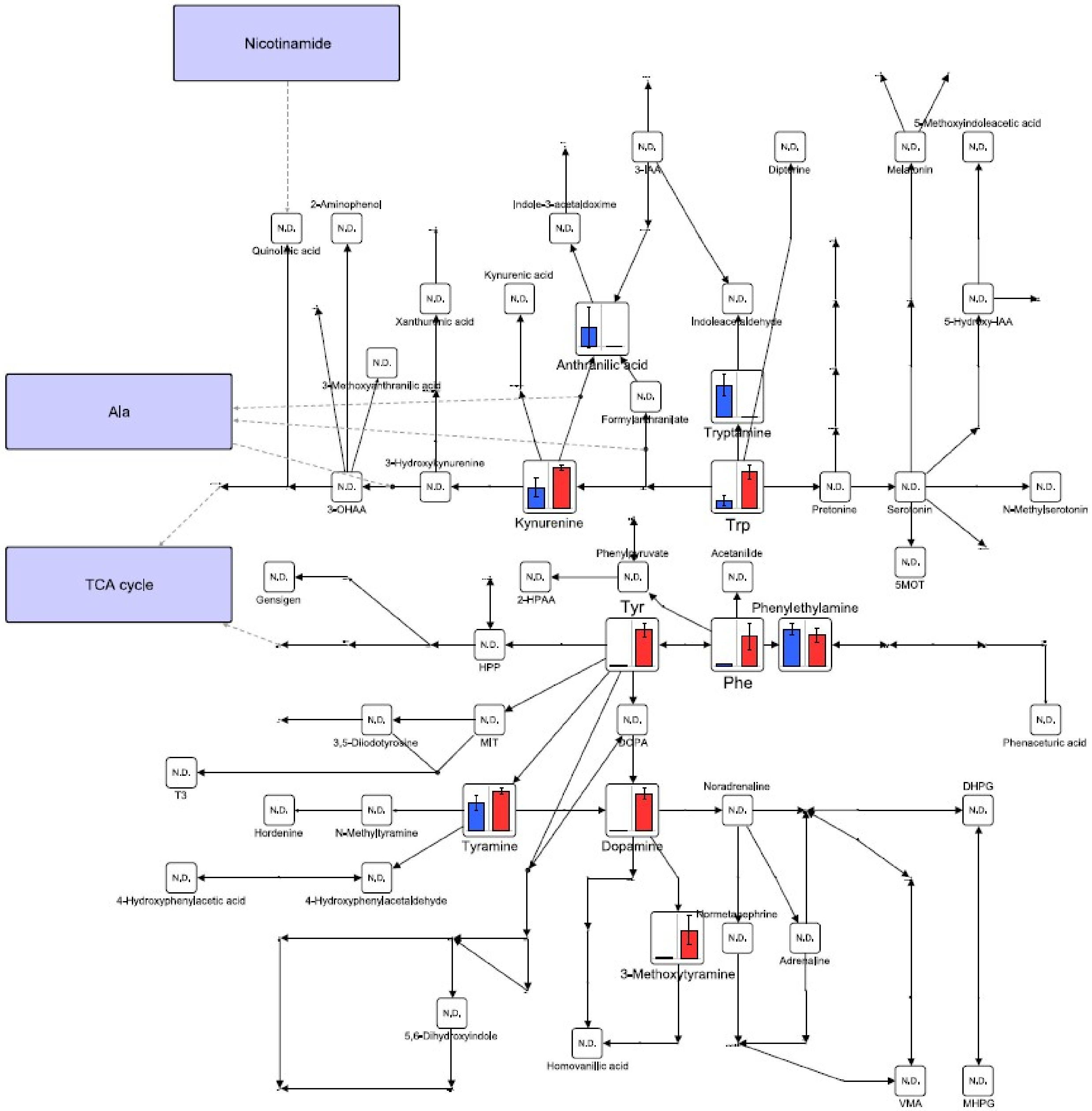

Figure S7. Pathway Map (Aromatic Amino Acids) In HMT Standard Metabolites, detected metabolites in this study are plotted on the pathway map. Blue and Red vertical bars show Control and Treatment, respectively. N.D.: Not Detected.

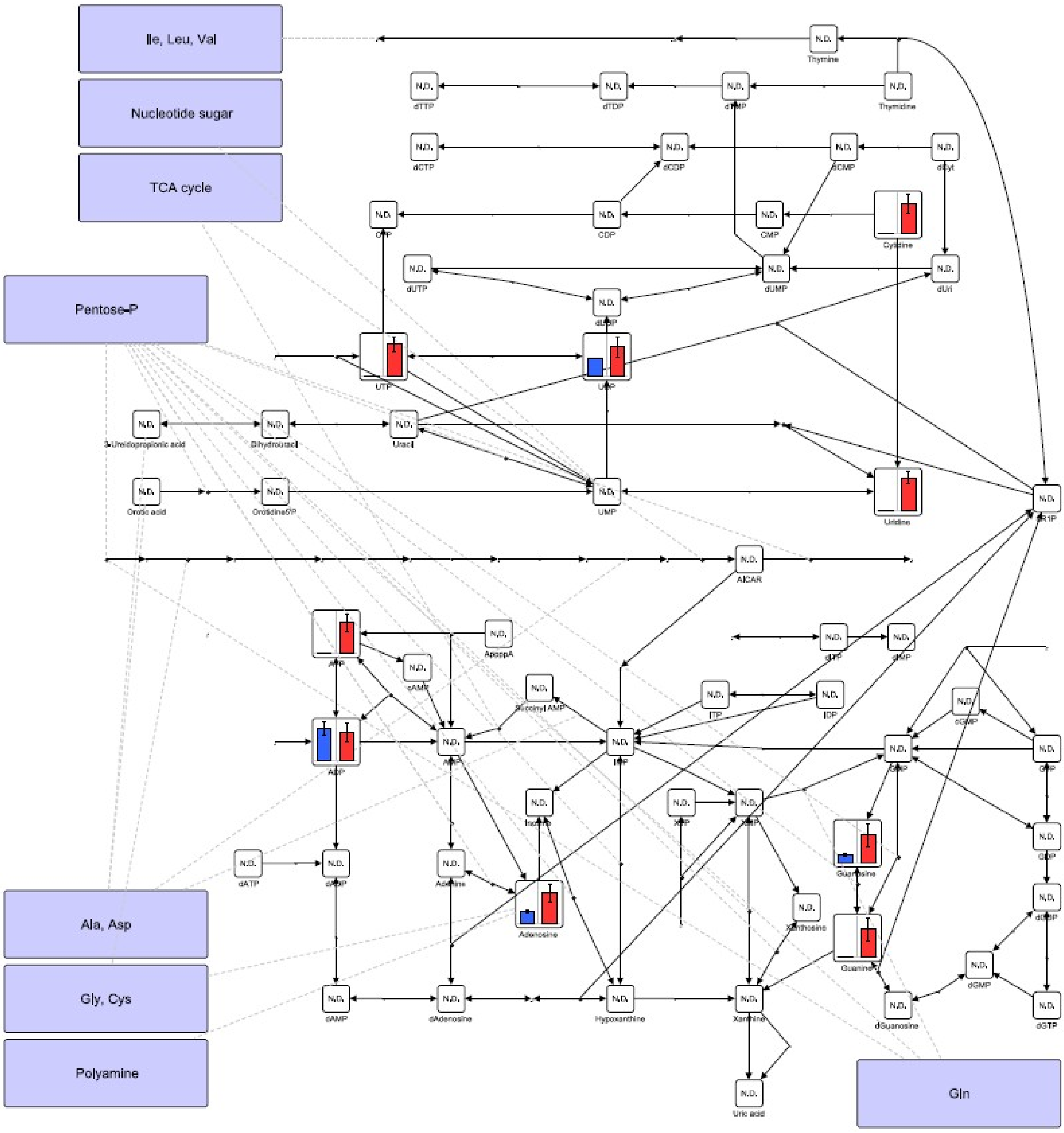

Figure S8. Pathway Map (Purines, Pyrimidines) In HMT Standard Metabolites, detected metabolites in this study are plotted on the pathway map. Blue and Red vertical bars show Control and Treatment, respectively. N.D.: Not Detected.

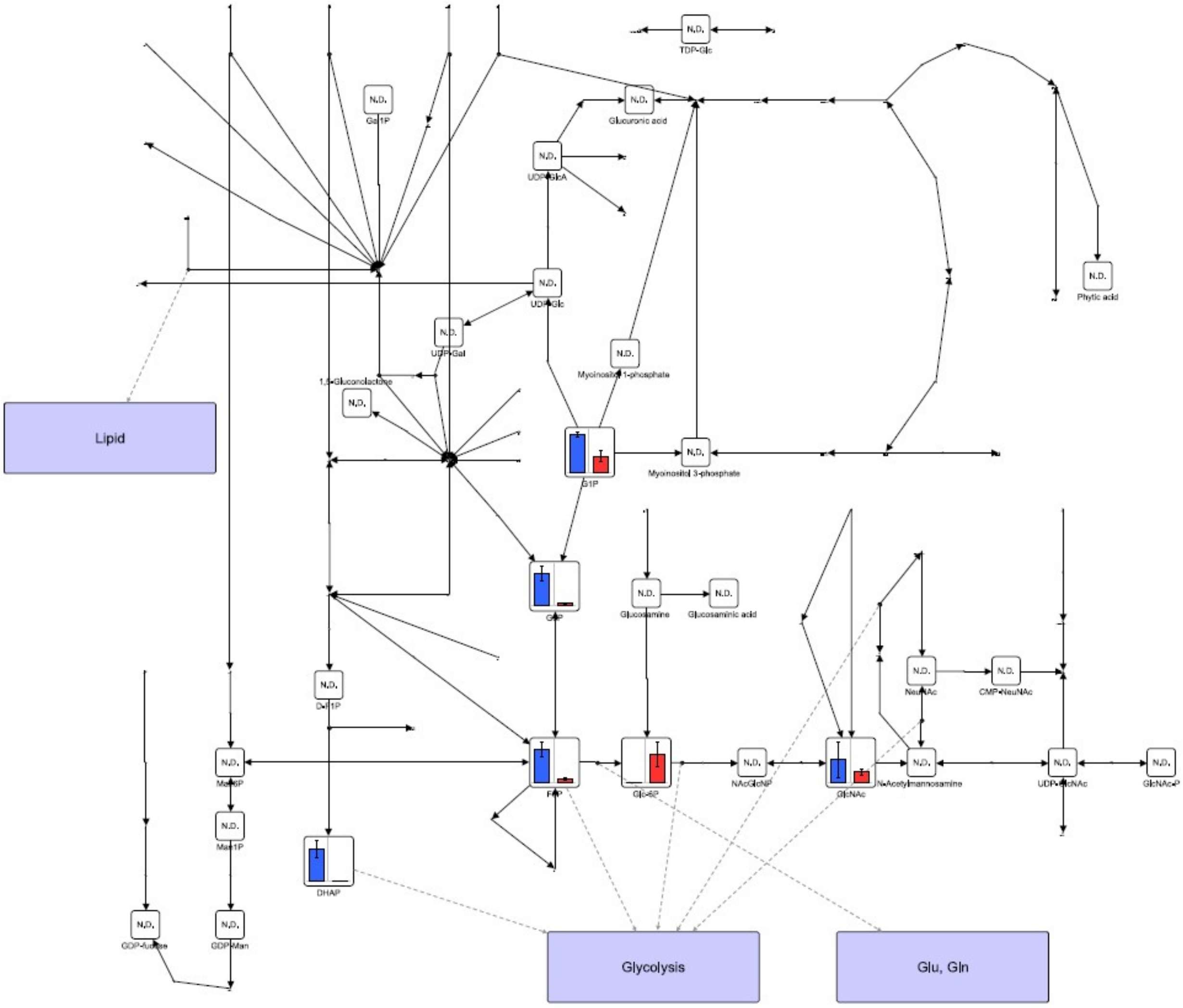

Figure S9. Pathway Map (Other Sugars) In HMT Standard Metabolites, detected metabolites in this study are plotted on the pathway map. Blue and Red vertical bars show Control and Treatment, respectively. N.D. : Not Detected.

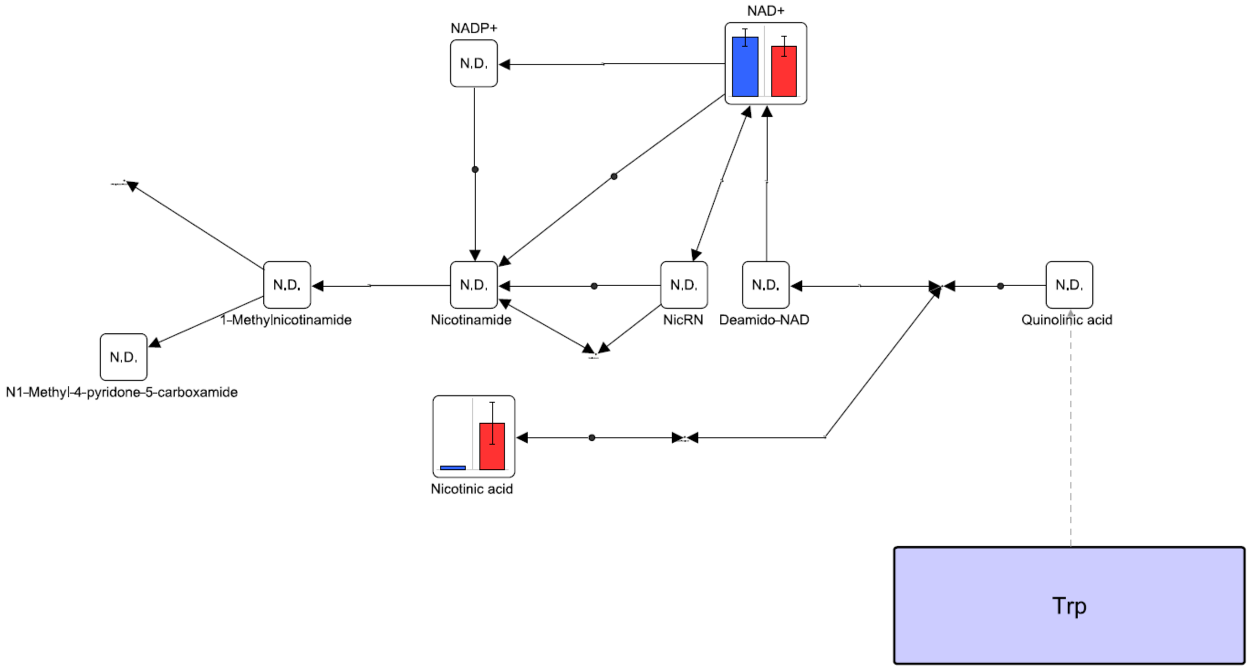

Figure S10. Pathway Map (Nicotinic Acid, Nicotinamide) In HMT Standard Metabolites, detected metabolites in this study are plotted on the pathway map. Blue and Red vertical bars show Control and Treatment, respectively. N.D. : Not Detected.

Table S1. Intracellular metabolites of glycolysis and pentose phosphate pathway detected from Control or *NbClpC1/C2* co-suppressed plants (ClpC) using CE-TOF-MS

ID	HMT DB† Compound name	m/z	MT					Comparative analysis	
				Control		ClpC		Control vs ClpC	
				Mean	Standard deviation	mean	Standard deviation	Ratio¶	*p*-value‖
A_0005	Lactic acid	89.025	11.123	0.001718	0.001240	0.002277	0.001184	1.3	0.603
A_0050	Sedoheptulose 7-phosphate	289.031	9.929	0.000059	N.A.	0.000031	N.A.	0.5	N.A.
A_0047	Fructose 6-phosphate	259.021	10.252	0.000465	0.000106	0.000047	0.000009	0.1	0.020*
A_0046	Glucose 6-phosphate	259.021	10.155	0.002120	0.000502	0.000171	0.000040	0.1	0.021*
A_0003	Pyruvic acid	87.008	13.427	0.000668	0.000027	N.D.	N.A.	<1	N.A.
A_0023	Dihydroxyacetone phosphate	168.990	13.136	0.000215	0.000059	N.D.	N.A.	<1	N.A.
A_0031	2-Phosphoglyceric acid	184.985	20.188	0.000202	0.000033	N.D.	N.A.	<1	N.A.
A_0032	3-Phosphoglyceric acid	184.985	20.627	0.001736	0.000523	N.D.	N.A.	<1	N.A.

ID consists of analysis mode and number. 'C' and 'A' showed cation and anion modes, respectively.

N.D.: Not Detected. The metabolite which was below detection limits.

N.A.: Not Available. The calculation was not possible.

†Putative metabolites which were assigned on the basis of m/z and MT

¶In ratio calculation, the latter was denominator.

‖*p*-value in Welch's t-test.*<0.05, **<0.01, ***<0.001

They were sorted by the ratio of ClpC to Control in descending order.

Table S2. Intracellular metabolites of TCA cycle detected from Control or *NbClpC1/C2* co-suppressed plants (ClpC) using CE-TOF-MS

ID	HMT DB† Compound name	m/z	MT					Comparative analysis	
				Control		ClpC		Control vs ClpC	
				Mean	Standard deviation	mean	Standard deviation	Ratio¶	*p*-value‖
A_0026	cis-Aconitic acid	173.009	30.440	N.D.	N.A.	0.000445	0.000003	1<	N.A.
A_0034	Citric acid	191.019	27.630	0.0180809	0.001779	0.306653	0.091248	17.0	0.032*
A_0012	Succinic acid	117.019	21.605	0.0003616	0.000027	0.001535	0.000396	4.2	0.035*
A_0010	Fumaric acid	115.003	27.065	0.0006934	0.000185	0.000416	0.0002	0.6	0.153
A_0016	Malic acid	133.014	21.698	1.0380618	0.247251	0.204184	0.138736	0.2	0.013*

ID consists of analysis mode and number. 'C' and 'A' showed cation and anion modes, respectively.

N.D.: Not Detected. The metabolite which was below detection limits.

N.A.: Not Available. The calculation was not possible.

†Putative metabolites which were assigned on the basis of m/z and MT

¶In ratio calculation, the latter was denominator.

‖*p*-value in Welch's t-test.*<0.05, **<0.01, ***<0.001

They were sorted by the ratio of ClpC to Control in descending order.

Table S3. Intracellular metabolites of glutamate, glutamine, histidine and proline metabolism detected from Control or *NbClpC1/C2* co-suppressed plants (ClpC) using CE-TOF-MS

ID	HMT DB† Compound name	m/z	MT					Comparative analysis	
				Control		ClpC		Control vs ClpC	
				Mean	Standard deviation	mean	Standard deviation	Ratio¶	*p*-value‖
C_0107	γ-Glu-Cys	251.069	12.947	N.D.	N.A.	0.000051	0.000023	1<	N.A.
A_0015	5-Oxoproline	128.034	9.840	0.000219	0.000049	0.03838	0.006133	175.4	0.008**
C_0084	Arg	175.118	7.206	0.0005294	0.000046	0.076976	0.014494	145.4	0.012*
C_0064	Gln	148.079	10.886	0.0006885	0.000216	0.037769	0.003193	54.9	0.002**
C_0070	His	156.076	7.391	0.0009947	0.000257	0.044087	0.004785	44.3	0.004**
C_0085	Citrulline	176.102	11.216	0.000069	0.000021	0.000946	0.00011	13.6	0.004**
C_0001	Urea	61.039	21.400	0.0013057	0.000385	0.007212	0.00256	5.5	0.054
C_0017	2-Aminobutyric acid	104.070	9.781	0.0001443	0.000036	0.000484	0.000269	3.4	0.158
C_0041	Imidazole-4-acetic acid	127.050	8.155	0.000023	0.000005	0.000061	0.000011	2.6	0.014*
C_0099	Spermine	203.222	4.538	0.0002004	0.000011	0.000478	0.000061	2.4	0.014*
C_0082	3-Methylhistidine	170.092	7.618	0.000065	0.000038	0.000153	0.000022	2.3	0.139
C_0046	N-Acetylputrescine	131.118	8.581	0.000038	0.000013	0.000078	0.000022	2.0	0.075
C_0116	Glutathione (GSSG)_divalent	307.082	12.340	0.0003861	0.000078	0.000776	0.000096	2.0	0.006**
C_0024	Histamine	112.087	4.843	0.000027	0.000002	0.000053	0.000007	2.0	0.021*
C_0086	Cys-Gly	179.047	9.349	0.000027	0.000004	0.000049	0.000008	1.8	0.029*
C_0063	Glu	148.060	11.086	0.0625691	0.006837	0.09708	0.010315	1.6	0.012*
C_0050	Ornithine	133.096	6.925	0.0001347	0.000088	0.00019	0.000074	1.4	0.458
C_0059	4-Guanidinobutyric acid	146.093	8.415	0.000133	0.000016	0.000149	0.000021	1.1	0.382
C_0117	Glutathione (GSH)	308.090	13.397	0.0108377	0.001288	0.011527	0.002917	1.1	0.735
C_0060	Spermidine	146.165	4.597	0.0021632	0.000433	0.001543	0.00013	0.7	0.122
C_0027	Pro	116.071	10.978	0.0556201	0.021324	0.038265	0.027243	0.7	0.437
C_0008	Putrescine	89.108	4.775	0.0007049	0.000244	0.000419	0.000052	0.6	0.176
C_0047	Hydroxyproline	132.066	12.214	0.0003885	0.000121	0.000081	0.000051	0.2	0.033*

ID consists of analysis mode and number. 'C' and 'A' showed cation and anion modes, respectively.

N.D.: Not Detected. The metabolite which was below detection limits.

N.A.: Not Available. The calculation was not possible.

†Putative metabolites which were assigned on the basis of m/z and MT

¶In ratio calculation, the latter was denominator.

‖*p*-value in Welch's t-test.*<0.05, **<0.01, ***<0.001

In Gln where peak intensity was saturated, relative area was calculated using ^{13}C data.

They were sorted by the ratio of ClpC to Control in descending order.

Table S4. Intracellular metabolites of glycine, serine and cysteine metabolism detected from Control or *NbClpC1/C2* co-suppressed plants (ClpC) using CE-TOF-MS

ID	HMT DB† Compound name	m/z	MT					Comparative analysis	
				Control		ClpC		Control vs ClpC	
				Mean	Standard deviation	mean	Standard deviation	Ratio¶	*p*-value‖
C_0022	Hypotaurine	110.027	18.327	0.0001178	0.00042	0.001236	0.000447	10.5	0.048*
C_0090	Phosphorylcholine	184.073	20.770	0.001488	0.000834	0.010589	0.00302	7.1	0.028*
C_0020	Choline	104.107	6.954	0.0043822	0.002807	0.025357	0.004999	5.8	0.007**
C_0021	Ser	106.050	10.120	0.0081975	0.00251	0.043741	0.015811	5.3	0.057
C_0032	Thr	120.065	10.634	0.0069943	0.000492	0.031652	0.008462	4.5	0.037*
C_0003	Gly	76.039	8.454	0.0017427	0.000295	0.007228	0.003265	4.1	0.099
C_0036	Cys	122.027	11.480	0.000184	0.000028	0.000719	0.000197	3.9	0.040*
C_0066	Met	150.059	10.825	0.0006122	0.000112	0.001014	0.000314	1.7	0.146
C_0028	Betaine	118.086	11.443	0.0004193	0.000052	0.000593	0.000106	1.4	0.086
A_0020	Citramalic acid	147.029	18.080	0.0009725	0.0003	0.0007	0.000237	0.7	0.288
C_0118	S-Adenosylmethionine	399.144	7.208	0.0002602	0.000015	0.000121	0.000016	0.5	0.000***
C_0109	Glycerophosphocholine	258.109	21.896	0.0066867	0.000466	0.001531	0.000309	0.2	0.000***
A_0025	Glycerol 3-phosphate	171.006	12.563	0.0003606	0.000059	0.000063	0.000008	0.2	0.012*
A_0002	Glycolic acid	75.009	13.136	0.0015659	0.000767	N.D.	N.A.	<1	N.A.

ID consists of analysis mode and number. 'C' and 'A' showed cation and anion modes, respectively.

N.D.: Not Detected. The metabolite which was below detection limits.

N.A.: Not Available. The calculation was not possible.

†Putative metabolites which were assigned on the basis of m/z and MT

¶In ratio calculation, the latter was denominator.

‖*p*-value in Welch's t-test.*<0.05, **<0.01, ***<0.001

They were sorted by the ratio of ClpC to Control in descending order.

Table S5. Intracellular metabolites of aspartate, alanine and lysine metabolism detected from Control or *NbClpC1/C2* co-suppressed plants (ClpC) using CE-TOF-MS

ID	HMT DB† Compound name	m/z	MT					Comparative analysis	
				Control		ClpC		Control vs ClpC	
				Mean	Standard deviation	mean	Standard deviation	Ratio¶	*p*-value‖
C_0113	Saccharopine	277.139	10.931	N.D.	N.A.	0.001109	0.000687	1<	N.A.
C_0075	2-Aminoadipic acid	162.075	11.086	0.000068	0.000011	0.004973	0.002308	72.8	0.067
C_0053	Asn	134.066	10.598	0.0001703	0.000099	0.011987	0.001024	70.4	0.002**
C_0061	Lys	147.112	6.976	0.0015157	0.000234	0.079861	0.020932	52.7	0.023*
C_0094	N6,N6,N6-Trimethyllysine	189.159	7.291	0.000057	0.000017	0.001834	0.000331	32.1	0.011*
C_0009	Ala	90.055	9.159	0.0077749	0.001197	0.049817	0.01258	6.4	0.028*
C_0043	Pipecolic acid	130.086	10.387	0.0002764	0.000122	0.001759	0.000544	6.4	0.037*
C_0052	Asp	134.044	11.732	0.0256789	0.006211	0.125632	0.018243	4.9	0.006**
C_0010	β-Ala	90.055	7.471	0.0004027	0.000133	0.000922	0.000063	2.3	0.010*
A_0008	3-Hydroxybutyric acid	103.040	9.838	0.0001567	0.000014	N.D.	N.A.	<1	N.A.

ID consists of analysis mode and number. 'C' and 'A' showed cation and anion modes, respectively.

N.D.: Not Detected. The metabolite which was below detection limits.

N.A.: Not Available. The calculation was not possible.

†Putative metabolites which were assigned on the basis of m/z and MT

¶In ratio calculation, the latter was denominator.

‖*p*-value in Welch's t-test.*<0.05, **<0.01, ***<0.001

In Asn where peak intensity was saturated, relative area was calculated using ^{13}C data.

They were sorted by the ratio of ClpC to Control in descending order.

Table S6. Intracellular metabolites of branched chain amino acid metabolism detected from Control or *NbClpC1/C2* co-suppressed plants (ClpC) using CE-TOF-MS

ID	HMT DB† Compound name	m/z	MT					Comparative analysis	
				Control		ClpC		Control vs ClpC	
				Mean	Standard deviation	mean	Standard deviation	Ratio¶	*p*-value‖
C_0051	Leu	133.105	10.411	0.000336	0.000053	0.014673	0.002021	43.7	0.007**
C_0030	Val	118.086	10.133	0.0071986	0.0016	0.146696	0.030952	20.4	0.016*
C_0049	Ile	132.101	10.334	0.0059692	0.000627	0.062732	0.008139	10.5	0.007**
C_0016	3-Aminoisobutyric acid	104.070	7.959	0.000064	0.000017	0.000486	0.000014	7.5	0.000***
A_0004	3-Hydroxypropionic acid	89.025	10.832	0.000146	0.000018	N.D.	N.A.	<1	N.A.

ID consists of analysis mode and number. 'C' and 'A' showed cation and anion modes, respectively.

N.D.: Not Detected. The metabolite which was below detection limits.

N.A.: Not Available. The calculation was not possible.

†Putative metabolites which were assigned on the basis of m/z and MT

¶In ratio calculation, the latter was denominator.

‖*p*-value in Welch's t-test.*<0.05, **<0.01, ***<0.001

In Leu where peak intensity was saturated, relative area was calculated using ^{13}C data.

They were sorted by the ratio of ClpC to Control in descending order.

Table S7. Intracellular metabolites of aromatic amino acid metabolism detected from Control or *NbClpC1*/*C2* co-suppressed plants (ClpC) using CE-TOF-MS

ID	HMT DB† Compound name	m/z	MT					Comparative analysis	
				Control		ClpC		Control vs ClpC	
				Mean	Standard deviation	mean	Standard deviation	Ratio¶	*p*-value‖
C_0069	Dopamine	154.087	8.826	N.D.	N.A.	0.000034	0.000005	1<	N.A.
C_0089	Tyr	182.080	11.516	0.0008257	0.000287	0.042575	0.008268	51.6	0.013*
C_0081	3-Methoxytyramine	168.101	8.977	0.000016	0.000002	0.000298	0.000146	18.1	0.079
C_0080	Phe	166.086	11.251	0.0020845	0.000201	0.028049	0.012886	13.5	0.073
C_0100	Trp	205.096	11.119	0.0011684	0.000748	0.005723	0.001081	4.9	0.006**
C_0101	Kynurenine	209.091	10.050	0.000021	0.000009	0.000041	0.000003	2.0	0.196
C_0058	Tyramine	138.091	8.405	0.0026671	0.000678	0.003784	0.000283	1.4	0.088
C_0038	2-Phenylethylamine	122.096	7.850	0.0198267	0.003413	0.016458	0.003948	0.8	0.327
C_0057	Anthranilic acid	138.055	10.613	0.0001873	0.00021	N.D.	N.A.	<1	N.A.
C_0073	Tryptamine	161.107	8.446	0.000059	0.000019	N.D.	N.A.	<1	N.A.

ID consists of analysis mode and number. 'C' and 'A' showed cation and anion modes, respectively.

N.D.: Not Detected. The metabolite which was below detection limits.

N.A.: Not Available. The calculation was not possible.

†Putative metabolites which were assigned on the basis of m/z and MT

¶In ratio calculation, the latter was denominator.

‖*p*-value in Welch's t-test.*<0.05, **<0.01, ***<0.001

They were sorted by the ratio of ClpC to Control in descending order.

Table S8. Intracellular metabolites of purine and pyrimidine metabolism detected from Control or *NbClpC1/C2* co-suppressed plants (ClpC) using CE-TOF-MS

ID	HMT DB† Compound name	m/z	MT					Comparative analysis	
				Control		ClpC		Control vs ClpC	
				Mean	Standard deviation	mean	Standard deviation	Ratio¶	*p*-value‖
A_0073	UTP	482.960	13.077	N.D.	N.A.	0.000107	0.000025	1<	N.A.
A_0075	ATP	505.986	12.210	N.D.	N.A.	0.000123	0.000035	1<	N.A.
C_0068	Guanine	152.056	8.424	N.D.	N.A.	0.000488	0.000201	1<	N.A.
C_0105	Cytidine	244.092	9.804	N.D.	N.A.	0.000122	0.000037	1<	N.A.
C_0106	Uridine	245.078	22.346	N.D.	N.A.	0.00018	0.000031	1<	N.A.
C_0115	Guanosine	284.098	12.719	0.000052	0.000007	0.000196	0.000083	3.7	0.095
C_0111	Adenosine	268.103	10.009	0.000024	0.000003	0.000063	0.000018	2.5	0.064
A_0064	UDP	402.993	12.076	0.000045	N.A.	0.000076	0.000026	1.7	N.A.
A_0068	ADP	426.020	11.240	0.000096	0.000018	0.000084	0.000027	0.9	0.585

ID consists of analysis mode and number. 'C' and 'A' showed cation and anion modes, respectively.

N.D.: Not Detected. The metabolite which was below detection limits.

N.A.: Not Available. The calculation was not possible.

†Putative metabolites which were assigned on the basis of m/z and MT

¶In ratio calculation, the latter was denominator.

‖*p*-value in Welch's t-test.*<0.05, **<0.01, ***<0.001

They were sorted by the ratio of ClpC to Control in descending order.

Table S9. Intracellular metabolites detected from Control or *NbClpC1*/*C2* co-suppressed plants (ClpC) using CE-TOF-MS which were not categorized on the basis of pathway metabolism

ID	HMT DB† Compound name	m/z	MT					Comparative analysis	
				Control		ClpC		Control vs ClpC	
				Mean	Standard deviation	mean	Standard deviation	Ratio¶	*p*-value‖
A_0018	Ethanolamine phosphate	140.011	8.254	N.D.	N.A.	0.000184	0.000019	1<	N.A.
A_0021	Phthalic acid	165.019	16.060	N.D.	N.A.	0.00015	N.A.	1<	N.A.
A_0029	2-Isopropylmalic acid	175.060	14.150	N.D.	N.A.	0.000617	0.000235	1<	N.A.
A_0043	Biotin	243.082	7.896	N.D.	N.A.	0.000096	0.000025	1<	N.A.
A_0044	Glucosamine 6-phosphate	258.037	8.507	N.D.	N.A.	0.000416	0.00019	1<	N.A.
C_0014	Homoserinelactone	102.055	7.138	N.D.	N.A.	0.000048	0.000014	1<	N.A.
C_0031	2,4-Diaminobutyric acid	119.081	6.957	N.D.	N.A.	0.000174	0.000025	1<	N.A.
C_0045	trans-Glutaconic acid	131.032	23.135	N.D.	N.A.	0.000066	N.A.	1<	N.A.
C_0065	Nornicotine	149.106	5.159	N.D.	N.A.	0.012213	0.001653	1<	N.A.
C_0071	Allantoin	159.050	22.287	N.D.	N.A.	0.016696	0.001296	1<	N.A.
C_0074	N6-Methyllysine	161.128	7.156	N.D.	N.A.	0.000225	0.000045	1<	N.A.
C_0087	S-Carboxymethylcysteine	180.032	13.206	N.D.	N.A.	0.000166	0.000044	1<	N.A.
C_0091	Gly-Leu	189.123	9.904	N.D.	N.A.	0.000086	0.000022	1<	N.A.
C_0092	N6-Acetyllysine	189.123	11.582	N.D.	N.A.	0.000342	0.000103	1<	N.A.
C_0093	Nω-Methylarginine	189.134	7.565	N.D.	N.A.	0.00127	0.000246	1<	N.A.
C_0097	ADMA	203.149	7.775	N.D.	N.A.	0.00072	0.000049	1<	N.A.
C_0098	SDMA	203.149	7.897	N.D.	N.A.	0.000178	0.000017	1<	N.A.
C_0110	Thiamine	265.111	6.691	N.D.	N.A.	0.000125	0.000035	1<	N.A.
C_0078	S-Methylmethionine	164.073	7.398	0.0002754	0.000044	0.023549	0.004685	85.5	0.013*
C_0039	Nicotinic acid	124.039	10.168	0.000027	N.A.	0.000401	0.000184	14.8	N.A.
C_0018	GABA	104.070	7.800	0.001047	0.000286	0.010309	0.003535	9.8	0.044*
C_0056	p-Aminobenzoic acid	138.055	9.884	0.000023	0.000009	0.000189	0.000041	8.1	0.014*
C_0002	Ethanolamine	62.061	6.460	0.0001613	0.000038	0.000853	0.000131	5.3	0.008**
C_0079	Methionine sulfoxide	166.052	11.968	0.0001042	0.000015	0.000438	0.000078	4.2	0.015*
C_0029	5-Aminovaleric acid	118.086	8.154	0.0006822	0.000391	0.002434	0.000374	3.6	0.005**
C_0083	N5-Ethylglutamine	175.107	11.530	0.0001323	0.000021	0.000426	0.000048	3.2	0.003**
C_0119	Cysteine glutathione disulfide	427.095	11.825	0.000024	0.000002	0.000054	0.000017	2.2	0.093
C_0072	Ala-Ala	161.091	9.393	0.000094	0.000043	0.000204	0.000056	2.2	0.061

(Continued)

ID	HMT DB† Compound name	m/z	MT					Comparative analysis	
				Control		ClpC		Control vs ClpC	
				Mean	Standard deviation	mean	Standard deviation	Ratio¶	*p*-value‖
C_0114	1-Methyladenosine	282.119	10.083	0.000054	0.000005	0.000113	0.000016	2.1	0.016*
C_0096	Gly-Asp	191.065	10.132	0.000058	0.000011	0.000109	0.000020	1.9	0.032*
C_0067	3-Methyladenine	150.077	8.048	0.0002704	0.000083	0.000496	0.000104	1.8	0.045*
C_0062	O-Acetylserine	148.060	12.802	0.0001691	0.000008	0.000308	0.000078	1.8	0.090
C_0023	Cytosine	112.051	7.356	0.000012	0.000002	0.000021	0.000023	1.7	0.011*
C_0037	2-Amino-2-(hydroxymethyl)-1,3-propanediol	122.080	8.431	0.000052	0.000022	0.000078	0.000017	1.5	0.194
A_0039	6,8-Thioctic acid	205.035	8.666	0.000048	0.000009	0.000070	0.000013	1.5	0.126
C_0005	Pyridine	80.049	5.990	0.0003372	0.000097	0.000468	0.000123	1.4	0.225
C_0077	Nicotine	163.122	5.367	0.023549	0.005349	0.032273	0.009866	1.4	0.269
A_0014	Benzoic acid	121.030	10.175	0.000071	0.000009	0.000092	0.000009	1.3	0.117
C_0012	1-Methyl-2-pyrrolidone	100.075	22.142	0.0002061	0.000028	0.000239	0.000009	1.2	0.175
C_0013	Cyclohexylamine	100.112	7.798	0.000095	0.000033	0.000107	0.000019	1.1	0.656
C_0042	Quinoline	130.066	7.302	0.000035	0.000001	0.000038	0.000005	1.1	0.439
A_0077	UDP-glucose<br>UDP-galactose	565.047	8.919	0.000424	0.000084	0.000369	0.000077	0.9	0.456
A_0078	NAD+	662.102	6.753	0.000061	0.000008	0.000052	0.000010	0.9	0.326
C_0044	Octylamine	130.158	8.574	70.000078	0.000026	0.000060	0.000011	0.8	0.359
C_0095	Castanospermine	190.107	9.124	0.000066	0.000026	0.000045	0.000008	0.7	0.470
A_0006	Malonic acid	103.003	34.499	0.0025821	0.000539	0.001588	0.000208	0.6	0.070
C_0076	Anabasine	163.122	5.555	0.0080292	0.00164	0.004828	0.000539	0.6	0.065
A_0048	myo-Inositol 1-phosphate<br>myo-Inositol 3-phosphate	259.021	10.601	0.0002141	0.000049	0.000104	0.000033	0.5	0.040*
C_0102	N-Acetylglucosamine	222.097	22.320	0.0007631	0.000593	0.000349	0.000097	0.5	0.349
A_0037	Gluconic acid	195.050	8.390	0.0024886	0.000341	0.001095	0.000202	0.4	0.007**
C_0006	Piperidine	86.097	7.056	0.0001214	0.000022	0.000053	0.000016	0.4	0.017*
A_0045	Glucose 1-phosphate	259.021	10.464	0.0002797	0.000020	0.000118	0.000041	0.4	0.009**
C_0108	Dyphylline	255.107	22.346	0.0004867	0.000068	0.000193	0.000031	0.4	0.009**
C_0004	Isopropanolamine	76.075	7.108	0.000057	0.000013	0.000019	0.000002	0.3	0.037*
A_0066	Trehalose 6-phosphate	421.079	8.771	0.0002636	0.000049	0.000065	0.000013	0.2	0.015*
A_0040	Ethyl glucuronide	221.066	7.746	0.0005006	0.000261	0.000098	N.A.	0.2	N.A.
C_0015	Hexylamine	102.127	7.902	0.0001793	0.000042	0.000033	0.000011	0.2	0.018*
A_0036	Galacturonic acid	193.033	8.603	0.0003693	0.000009	0.000067	0.000022	0.2	0.000***

(Continued)

ID	HMT DB[†] Compound name	m/z	MT					**Comparative analysis**	
				**Control**		**ClpC**		**Control vs ClpC**	
				**Mean**	**Standard deviation**	**mean**	**Standard deviation**	**Ratio**[¶]	***p*-value**[‖]
A_0030	N-Formylmethionine	176.040	8.289	0.2241575	0.009903	0.02858	0.006372	0.1	0.000***
C_0088	Theobromine	181.072	22.307	0.0067163	0.001028	0.000676	0.000034	0.1	0.009**
A_0027	Shikimic acid	173.044	8.458	0.0010289	0.000075	0.000084	0.000016	0.1	0.001**
A_0009	Glyceric acid	105.019	10.746	0.0155474	0.006989	0.000747	0.000563	0.0	0.066
A_0028	Ascorbic acid	175.024	8.784	0.0277005	0.007734	0.000911	0.000367	0.0	0.026*
A_0017	Threonic acid	135.029	9.628	0.1029809	0.03361	0.003258	0.002641	0.0	0.035*
A_0035	Quinic acid	191.055	8.591	0.3002112	0.021775	0.001199	0.000826	0.0	0.002**
A_0013	2-Hydroxyvaleric acid	117.055	9.292	0.0002017	0.000078	N.D.	N.A.	<1	N.A.
C_0033	2-Methylserine	120.065	10.520	0.000038	0.000011	N.D.	N.A.	<1	N.A.
A_0018	Ethanolamine phosphate	140.011	8.254	N.D.	N.A.	0.000184	0.000019	1<	N.A.

ID consists of analysis mode and number. 'C' and 'A' showed cation and anion modes, respectively.

N.D.: Not Detected. The metabolite which was below detection limits.

N.A.: Not Available. The calculation was not possible.

[†]Putative metabolites which were assigned on the basis of m/z and MT

[¶]In ratio calculation, the latter was denominator.

[‖]*p*-value in Welch's t-test.*<0.05, **<0.01, ***<0.001

They were sorted by the ratio of ClpC to Control in descending order.
